# Elucidating tumor heterogeneity from spatially resolved transcriptomics data by multi-view graph collaborative learning

**DOI:** 10.1038/s41467-022-33619-9

**Published:** 2022-10-10

**Authors:** Chunman Zuo, Yijian Zhang, Chen Cao, Jinwang Feng, Mingqi Jiao, Luonan Chen

**Affiliations:** 1 Institute of Artificial Intelligence, Donghua University, Shanghai, 201620 China; 2 Key Laboratory of Systems Biology, Shanghai Institute of Biochemistry and Cell Biology, Center for Excellence in Molecular Cell Science, Chinese Academy of Sciences, Shanghai, 200031 China; 3 Department of General Surgery, Xinhua Hospital Affiliated to Shanghai Jiao Tong University School of Medicine, Shanghai, 200092 China; 4 School of Biomedical Engineering and Informatics, Nanjing Medical University, Nanjing, 211166 China; 5 Key Laboratory of Information Fusion Technology of Ministry of Education, School of Automation, Northwestern Polytechnical University, Xi’an, 710072 China; 6 Key Laboratory of Systems Health Science of Zhejiang Province, School of Life Science, Hangzhou Institute for Advanced Study, University of Chinese Academy of Sciences, Chinese Academy of Sciences, Hangzhou, 310024 China; 7 Guangdong Institute of Intelligence Science and Technology, Hengqin, Zhuhai, Guangdong 519031 China; 8 School of Life Science and Technology, ShanghaiTech University, Shanghai, 201210 China

**Keywords:** Bioinformatics, Computational models, Data integration, Cancer genomics, Tumour heterogeneity

## Abstract

Spatially resolved transcriptomics (SRT) technology enables us to gain novel insights into tissue architecture and cell development, especially in tumors. However, lacking computational exploitation of biological contexts and multi-view features severely hinders the elucidation of tissue heterogeneity. Here, we propose stMVC, a multi-view graph collaborative-learning model that integrates histology, gene expression, spatial location, and biological contexts in analyzing SRT data by attention. Specifically, stMVC adopting semi-supervised graph attention autoencoder separately learns view-specific representations of histological-similarity-graph or spatial-location-graph, and then simultaneously integrates two-view graphs for robust representations through attention under semi-supervision of biological contexts. stMVC outperforms other tools in detecting tissue structure, inferring trajectory relationships, and denoising on benchmark slices of human cortex. Particularly, stMVC identifies disease-related cell-states and their transition cell-states in breast cancer study, which are further validated by the functional and survival analysis of independent clinical data. Those results demonstrate clinical and prognostic applications from SRT data.

## Abstract (web summary)

Multi-view graph approaches could enhance the analysis of tissue heterogeneity in spatial transcriptomics. Here, the authors develop the Spatial Transcriptomics data analysis by Multiple View Collaborative-learning - stMVC - framework, and apply it to detect spatial domains and cell states in brain and tumor tissues.

## Introduction

The recent technological innovation of SRT platform, including sequencing-based technology (e.g., 10X Genomics Visium and Stereo-seq) and imaging-based technology (e.g., STARmap)^1–3^ allows profiling gene expression patterns in the spatial contexts of tissue^4^. These resulting multiple types of profiles: histology, spatial location, and gene expression, provide novel insights into the organization of cells and developmental biology, especially for the evolution of the tumor^5–7^. However, SRT data analysis for biology discovery remains challenging due to its low throughput, little sensitive, much sparse, and noisy^8,9^.

Recently, several computational methods have been designed to analyze SRT data^8^. For example, Giotto uses a similar processing strategy to single-cell RNA-seq (scRNA-seq), for feature selection, dimension reduction, and unsupervised clustering^10^. BayesSpace utilizes a fully Bayesian statistical method to enhance the spatial measurement via spatial neighborhood structure for clustering analysis^9^. SpaGCN adopting a graph convolutional network (GCN) approach integrates gene expression, spatial location, and histology to identify spatial domains and spatially variable genes (SVGs)^11^. stLearn integrates features of histology with spatial location to normalize gene expression data, and followed by clustering^12^. Squidpy brings together omics and image analysis tools to enable scalable description of spatial transcriptomics and proteomics data^13^. ClusterMap incorporates physical location and gene identity of RNAs to identify biologically meaningful structures from image-based in situ transcriptomics data^14^. DR-SC^15^ and SC-MEB^16^ utilizing latent hidden Markov random field model integrates gene expression and spatial location for spatial clustering. STAGATE^17^ combines gene expression and spatial information to detect spatial domains via graph attention auto-encoder framework. While these methods have many interesting findings, the lack of visual features that can be effectively and globally extracted from histology, efficient multi-view information fusion, and the biological contexts such as global positional information within a tissue, limits their disentangling capabilities in developmental biology.

On the other hand, GCN-based models have appeared as powerful tools to learn the representations of scRNA-seq data (i.e., by scGNN)^18^ and SRT data (i.e., by SpaGCN)^11^, however, these methods usually study networks with a single type of proximity between nodes, namely single-view network. Although SpaGCN proposes an RGB color space averaging strategy to convert histological data into the same measurement space as 2D space in the tissue slice as a third dimension before calculating the similarity between any two spots, to a certain extent, this strategy discards the texture features in each spot, i.e., the strategy extracts the color features from color space without fully utilizing the spatial distribution of gray tone variations within a specified area namely texture features^19^. Besides, in SRT studies, $K$-nearest spots that are physically closest to the central spot are not necessarily the same as those that are the most histologically similar to the spot, and the distance evaluation metrics between multi-view data are also not the same, thus yielding networks with multiple views. Moreover, the contributions of neighboring spots to determine the cell type to which the central spot belongs are not identical, which is consistent with the assumption of graph attention network (GAT)^20,21^. More importantly, the quality of the information in different views may be different, hence, it would be preferable that one novel model can learn the representations for each view by GAT, and meanwhile collaboratively integrate multiple networks to learn robust representations by automatically training the weights of different views^22^.

We reason that (i) cells belonging to the same cell type but distributed in different areas and interacting with different cell types in the tissue may have different cell-states^8^; (ii) the determination of each cell type (or cell-state) to which each cell belongs, is related to its size, shape, and arrangement (i.e., tightness or looseness), hence the texture data of histology has rich information to characterize cell type or cell-state^23^; and (iii) the colors of the antibodies on the immunofluorescence staining of the tumor sample can roughly mark the tumor position in the tissue, yielding region segmentation indicating biological contexts related to the tumor development.

In this work, we introduce stMVC (**S**patial **T**ranscriptomics data analysis by **M**ultiple **V**iew **C**ollaborative-learning), a framework that integrates four-layer information to elucidate tissue heterogeneity by attention-based multi-view graph collaborative learning, i.e., histology, gene expression data, spatial location, and region segmentation (e.g., tumor position) indicating biological contexts. The features of stMVC are as follows: (i) for each spot, globally learning efficient visual features while removing artifacts from histology by data augmentations and contrastive learning; (ii) learning robust representations for each spot by training the weights of multi-view graphs including histological-similarity-graph (HSG) by visual features and spatial-location-graph (SLG) by physical coordinates, via attention-based collaborative learning strategy, under semi-supervision of region segmentation; (iii) for samples of human ovarian endometrial adenocarcinoma (OEAD) and breast invasive ductal carcinoma (IDC), identifying cancer-related cell-states (i.e., stemness, migration, and metastasis) missed by competing methods, and also transition cell-states, which are further validated by clinical data from other independent studies, demonstrating potential clinical and prognostic applications from SRT data; and (iv) for sample of mouse primary visual cortex, enabling us to detect layer-specific inhibitory neurons. In particular, such a multi-view graph collaborative learning method is a flexible framework that is able to integrate not only SRT data from multi-sources or spatial multi-omics data but also spatial epigenomics or proteomics data.

## Results

### Overview of stMVC model

**Fig. 1:**
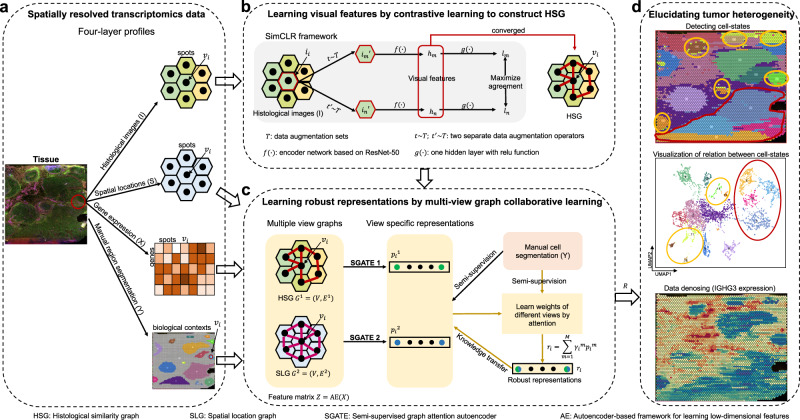
Overview of stMVC model. **a** Given each SRT data with four-layer profiles: histological images ($I$), spatial locations ($S$), gene expression ($X$), and manual region segmentation ($Y$) as the input, stMVC integrates them to disentangle tissue heterogeneity, particularly for the tumor. **b** stMVC adopts SimCLR model with feature extraction framework from ResNet-50 to efficiently learn visual features (${h}_{i}$) for each spot (${v}_{i}$) by maximizing agreement between differently augmented views of the same spot image (${i}_{i}$) via a contrastive loss in the latent space (${l}_{i}$), and then constructs HSG by the learned visual features ${h}_{i}$. **c** stMVC model adopting SGATE model learns view-specific representations (${{p}_{i}}^{1}$ and ${{p}_{i}}^{2}$) for each of two graphs including HSG and SLG, as well as the latent feature from gene expression data by the autoencoder-based framework as a feature matrix, where a SGATE for each view is trained under weak supervision of the region segmentation to capture its efficient low-dimensional manifold structure, and simultaneously integrates two-view graphs for robust representations (${r}_{i}$) by learning weights of different views via attention mechanism. **d** Robust representations $R$ can be used for elucidating tumor heterogeneity: detecting spatial domains, visualizing the relationship distance between different domains, and further denoising data.

stMVC collaboratively integrates histological image ($I$), spatial locations ($S$), and gene expression data ($X$), through the semi-supervision learning from biological contexts (i.e., region segmentation, $Y$, see Manual region segmentation) within a tissue, to clarify tissue heterogeneity (Fig. 1a–d). Specifically, we (1) learned the visual features (${h}_{i}$) for each spot image (${i}_{i}$) by ResNet-50 model^24^ (an efficient computer vision framework) that was trained by maximizing the agreement between differently augmented views of the same histological spot image, and then constructed HSG based on ${h}_{i}$ (Fig. 1b); and (2) captured the spot proximities encoded in either histology or spatial location, as well as the low-dimensional features (${z}_{i}$) from gene expression data by autoencoder (AE) (see Supplementary Methods), and then simultaneously integrated these two graphs for the robust representations (${r}_{i}$) by automatically learning the weight of view-specific representations (${{p}_{i}}^{1}$ and ${{p}_{i}}^{2}$) from two graphs via attention, under semi-supervision of region segmentation (Fig. 1c). Hence, $R$ is a low-dimensional feature representing the variation of these four-layer profiles, which is used to represent each spot as a point in the low-dimensional space, for spatial clustering and visualization, where each cluster is considered as a spatial domain. Subsequently, for each spot, we denoised gene expression data by its 15 nearest neighboring spots that are calculated based on the distance of robust representations, and then identified SVGs that are over-expressed in a domain by differential expression analysis (Fig. 1d).

To emphasize the advantages of stMVC, we designed several comparative methods in our experiments: (i) the mean of all view-specific representations was used as a comparison to assess the efficiency of the attention-based multi-view integration strategy enabling the network to focus on the key features for characterizing tissue structure, which is named stMVC-M; (ii) the single-view graph representations by semi-supervised graph attention autoencoder (SGATE) for either SLG (SGATE-SLG) or HSG (SGATE-HSG) were utilized to assess the effectiveness of the robust representations by multi-view graphs; (iii) the naïve HSG (SGATE-HSG-N) constructed based on the visual features by the ResNet-50 model (pre-trained by ImageNet^25^) was used to evaluate the quality of the visual features by our ResNet-50 model trained by histological images; and (iv) the low-dimensional representations encoded from gene expression data, via AE or semi-AE extended from AE through adding a classifier on bottleneck layer (see Supplementary Methods), were used to check if or not the components of semi-supervised learning and graph attention mechanism used by SGATE-SLG are responsible for capturing complex structure of SRT data.

### stMVC reveals the trajectory relationship between different spatial domains

**Fig. 2:**
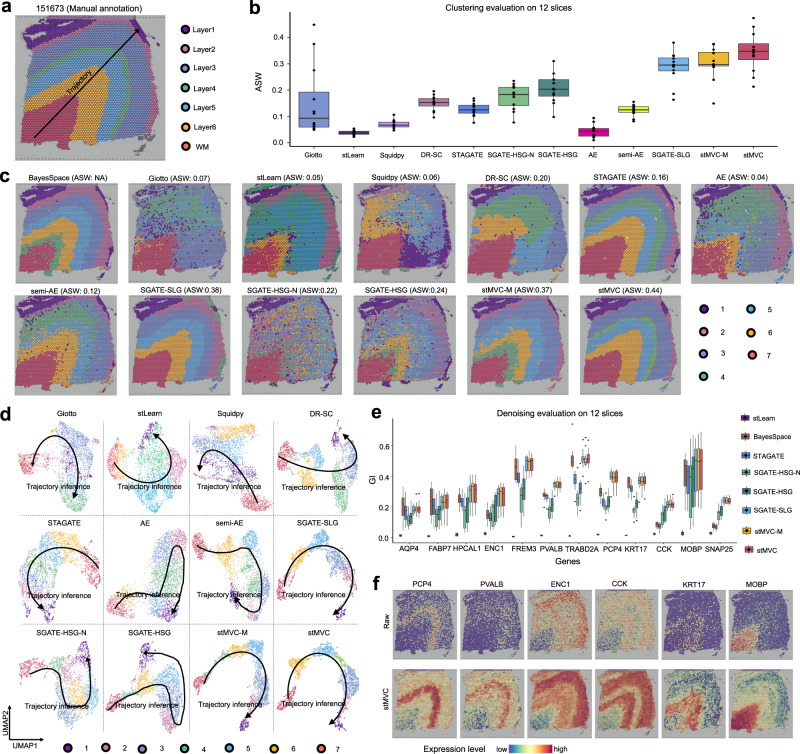
stMVC is able to detect spatial domains, visualize the relationship distance between different domains, and denoise data on the DLPFC dataset. **a** Annotation of seven DLPFC layers in slice 151673 by the previous study^26^, and the spatial adjacency and chronological order between these layers are WM → Layer6 → Layer5 → Layer4 → Layer3 → Layer2 → Layer1. **b** Boxplot of clustering accuracy in terms of average silhouette width (ASW) for assessing the closeness of multi-view joint features of same cluster compared to the other clusters, on all *n* = 12 samples. In the boxplot, the center line, box limits and whiskers separately indicate the median, upper and lower quartiles and 1.5 × interquartile range. **c** Spatial domains detected by BayesSpace, Giotto, stLearn, Squidpy, DR-SC, STAGATE, AE, semi-AE, the three SGATE-based single-view models, stMVC-M, and stMVC on slice 151673. **d** Scatter plot of the two-dimensional UMAP extracted from the latent features by Giotto, stLearn, Squidpy, DR-SC, STAGATE, AE, semi-AE, three SGATE-based single-view models, stMVC-M, and stMVC on slice 151673. Note that the trajectory between seven layers or domains is consistent with (**a**), and for each method, the predicted clusters and their colors are the same as (**c**). Note that subplot **b**–**d** BayesSpace cannot calculate ASW and visualized using UMAP. **e** Boxplot of the Gini-index (GI) score of gene expression data denoised by stLearn, BayesSpace, STAGATE, the three SGATE-based single-view models, stMVC-M, and stMVC for *n* = 12 samples. The higher GI score, the better the denoised data. In the boxplot, the center line, box limits and whiskers indicate the median, upper and lower quartiles and 1.5 × interquartile range, respectively. **f** Spatial expression of layer-specific genes:^26^ *PCP4*, *PVALB*, *ENC1*, *CCK*, *KRT17*, and *MOBP* for slice 151673 data denoised by stMVC, where we also provide raw data as a comparison. Source data are provided as a Source Data file.

The most important feature of the stMVC model is that the learned low-dimensional representations can reveal the trajectory relationship between different spatial domains through semi-supervised biological contexts. To assess the performance of stMVC, we applied it to process 12 slices of the human dorsolateral prefrontal cortex (DLPFC) dataset, each of which was manually annotated with six layers and white matter (WM), and the spatial adjacency and chronological order among these layers are WM → Layer6 → Layer5 → Layer4 → Layer3 → Layer2 → Layer1 (Fig. 2a)^26,27^. By default, through the semi-supervised learning of 70% of the annotation (Supplementary Fig. 1, Evaluation of proportion of labels for model training in Supplementary Note 1), stMVC, stMVC-M, and the three SGATE-based single-view models extracted 10-dimensional features from the input. For comparison, we also applied six recently developed methods including BayesSpace, Giotto, stLearn, Squidpy, DR-SC, and STAGATE for spatial clustering or visualization with the default parameters. Subsequently, we predicted cell clusters by the Louvain algorithm, evaluated clustering accuracy including ROGUE^28^ and average silhouette width (ASW)^29^ by calculating the similarity of the transcriptome and multi-view joint features between spots within each predicted cell cluster, respectively, and then visualized these low-dimensional features by mapping them into two uniform manifold approximation and projection (UMAP) spaces.

In summary (Fig. 2b–d and Supplementary Figs. 2–4), we found that (i) the clustering accuracy of stMVC is higher than that of the three SGATE-based single-view models, AE, semi-AE, BayesSpace, Giotto, stLearn, Squidpy, DR-SC, and STAGATE, among which ASW for the joint features is the highest while ROGUE for the transcriptomics is similar to other tools, indicating that our multi-view graph collaborative learning model did capture more useful information beyond transcriptomics data than that from single-view graph model; (ii) stMVC performs better than stMVC-M, especially in detecting the precise structure, e.g., Layer4 and Layer6 on slice 151673, demonstrating that the attention-based multi-view integration strategy enables the network to capture critical features for clarifying tissue structure, compared to those by the mean-based strategy; (iii) SGATE-HSG performs better than SGATE-HSG-N, which indicates that our trained visual features extraction model adopted from ResNet-50 by data augmentations and contrastive learning did learn more rich visual features than those from naïve ResNet-50 model pre-trained by ImageNet; (iv) SGATE-SLG performs better than semi-AE and AE, while AE performs worst, indicating that semi-supervised learning and graph attention mechanism are considered responsible for capturing data structure; and (v) each spatial domain is assigned almost by different feature embeddings from stMVC, stMVC-M, and SGATE-SLG model, compared to Giotto, stLearn, Squidpy, DR-SC, STAGATE, AE, semi-AE, and the two HSG-based models, and the distance between different domains of stMVC can reflect the trajectory of chronological order, which shows that stMVC can clarify the complex relationship between different spatial domains.

We further assessed the quality of the denoised data from stMVC by our defined Gini-index (GI) scores based on how far the expression distribution of layer-specific genes deviated from a totally equal distribution, where the higher the GI score, the more layer-specific the distribution of gene expression (see Evaluation of denoising of gene expression data). Overall, we observed that stMVC, stMVC-M, and SGATE-SLG have comparable performance, and those three methods perform better than BayesSpace, STAGATE and the two HSG-based models, while stLearn performs worst. For instance, some known layer marker genes such as *PCP4*, *PVALB*, *ENC1*, *CCK*, *KRT17*, and *MOBP*^26^ are more specifically expressed in their corresponding spatial location on slice 15,1673, compared with those by BayesSpace and STAGATE (Fig. 2e, f and Supplementary Fig. 5).

### stMVC contributes to detecting cell-states missed by competing methods

**Fig. 3:**
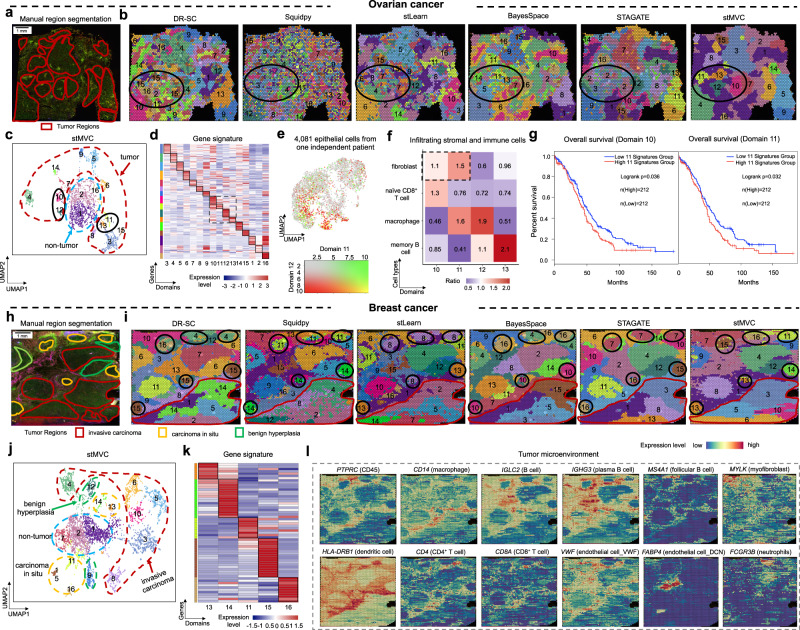
stMVC enables the detection of cell-states distributed in different spatial domains on ovarian and breast cancers. **a** Immunofluorescent staining of the tissue section and 17 manually segmented tumor regions. The intensity of DAPI, cytokeratin, and CD45 is shown in blue, green, and yellow. **b** Spatial clustering by DR-SC, Squidpy, stLearn, BayesSpace, STAGATE, and stMVC. **c** UMAP visualization of latent features by stMVC. Each domain is outlined by the region it belongs to, i.e., tumor or non-tumor. **d** Heatmap of average gene expression of signature genes from 16 domains by stMVC. Rows and columns indicate genes and domains. **e** UMAP plot of average expression of signature genes for domains 11 and 12 in scRNA-seq data of 4081 epithelial cells from one independent ovarian cancer. **f** Enrichment of infiltrating stromal and immune cells in each domain compared to the total distribution of those in four domains. The ratio is calculated by chi-square test^85^. The larger the ratio, the more cells are enriched in the domain. **g** Total survival rate of patients with the high ($>$ median value) or low (low $<$ median value) expression level of 11 signature genes for domains 10 and 11 in gene expression data of ovarian cancer from TCGA by GEPIA2^86^ (Supplementary Table 1). **h** Immunofluorescent staining of the tissue section and 15 manually segmented tumor regions, where those regions are outlined by their histological annotations: invasive carcinoma (red), carcinoma in situ (yellow), and benign hyperplasia (green). The intensity of DAPI, fiducial frame, and anti-CD3 is shown in green, blue, and yellow. **i** Spatial clustering by DR-SC, Squidpy, stLearn, BayesSpace, STAGATE, and stMVC. **j** UMAP visualization of latent features by stMVC. Domains are outlined by their histological annotations. **k** Heatmap of gene expression of signature genes from five domains enriched in the carcinoma in situ region by stMVC. Rows and columns indicate genes and domains. **l** Spatial expression of classical marker genes for stromal and immune cells, including *PTPRC*, *CD14*, *IGCL2*, *IGHG3*, *MS4A1*, *MYLK*, *HLA*-*DRB1*, *CD4*, *CD8A*, *ENG*, *FABP4*, and *FCGR3B* for data denoised by stMVC. Source data are provided as a Source Data file.

To illustrate that stMVC is able to clarify cancer cells that are distributed at different positions in the tissue, we further analyzed ovarian cancer (i.e., OEAD) and breast cancer (i.e., IDC) publicly published by 10X Genomics. We separately annotated 18 and 16 regions for ovarian and breast cancer based on our segmentation strategy detailed in Manual region segmentation, where for breast cancer, 15 tumor regions were classified into three different types: invasive carcinoma, carcinoma in situ, and benign hyperplasia by the previous study^9^ (Fig. 3a, h and Supplementary Figs. 6a and 8a). We treated the annotated regions as rough labels, and randomly selected 70% of them to supervise the training of the stMVC. stMVC, stMVC-M, and the three SGATE-based single-view models extracted 18- and 16- dimensional features from ovarian and breast cancer, respectively. Subsequently, we predicted cell clusters by the Louvain algorithm and visualized these low-dimensional features by mapping them into two-dimensional UMAP. For comparison, we also applied Giotto, DR-SC, Squidpy, stLearn, BayesSpace, and STAGATE for spatial clustering or visualization.

We found that stMVC facilitates detecting more domains enriched for the cancer regions, compared to those by all other computational methods (Fig. 3b–d, i–k and Supplementary Figs. 6b, c, 7, 8b, c and 9). Particularly for the regions outlined with black color on the ovarian cancer, we noted that stMVC detects four domains while other methods detect one or two domains, and also for the regions marked in black on the breast cancer, stMVC detects five regions while other methods detect at most three regions. Additionally, the feature embeddings extracted by stMVC are better separated between those different domains than those by Squidpy, Giotto, DR-SC, stLearn, and STAGATE, and each domain has its specific signature genes named SVGs.

To further support the accuracy of detecting cell-states missed by other methods, e.g., four domains of ovarian cancer, we adopted three independent ways. Specifically, we found that (i) the domains 11 and 12 cells are enriched in different cell populations that are not clearly separated in the scRNA-seq data of 4081 epithelial cells of one additional ovarian cancer sample from previous research^30^, by evaluating whether or not the average expression levels of the signature genes between two domains are different or not correlated (Fisher’s exact test, $p=0.2377$, Fig. 3e, see Supplementary Methods), indicating that these cells exist in ovarian cancer, as well as the advantage of SRT data over scRNA-seq data in terms of visualization of the gene expression levels in the context of tissue; (ii) four domains have different functions, which may be influenced by the infiltrating stromal and immune cells (Fig. 3f and Supplementary Fig. 10a, b, Estimation of cell populations for each spot by SpatialDecon^31^ in Supplementary Methods), i.e., domain 10 cells are involved in TNF-$\alpha$ by infiltrating immune cells such as CD8^+^ T cell^32^ to elevate NF-kB activity for increasing the risk of cancer, and modulating IL-6/STAT3 signaling to create a positive feedback loop for cell proliferation and cancer initiation;^33^ domain 11 cells are related to HIF-1 signaling pathway induced by infiltrating cancer-associated fibroblasts (CAFs)^34^, and NRF2 pathway for tumor adaptation to microenvironmental hypoxia^35^; domain 12 cells show overexpression of matrix metalloproteinases genes^36^, and TGF-beta secreted by infiltrating macrophages^37^, for promoting ovarian cancer metastasis and migration; and domain 13 cells are mediated by VEGFA/VEGFR2 signaling pathway serving a vital function in the angiogenesis of ovarian cancer;^38^ and (iii) the expression of signature genes of domains 10 ($p=0.036$) and 11 ($p=0.032$) with more enriched CAFs is significantly correlated with shorter overall survival, which was evaluated by an independent ovarian cancer dataset from the TCGA database (Fig. 3g and signature genes in Supplementary Table 1). The conclusion is consistent with the previous research that the proportion of CAF in ovarian cancer is associated with a poor prognosis^39^. In addition, we noted that domain 13 cells exhibit over-expression of *NEDD9* which is associated with the progression of and an unfavorable prognosis in ovarian cancer^40^.

In addition to infiltrating stromal and immune cells, we found that distinct cell-states are surrounded by different microenvironments. For example, among the five domains in the region of carcinoma in situ of breast cancer (Fig. 3i–l and Supplementary Fig. 8d, e), we noted that domains 13 and 14 are surrounded by more immune cells such as macrophage (*CD14*), dendritic cell (*HLA-DRB1*), and T cell (*CD4* and *CD8A*); domains 11, 15, and 16 are mostly surrounded by plasma B cell (*IGCL2* and *IGHG3*) and two types of endothelial cell (*FABP4* for endothelial cell_DCN, *VWF* and *ENG* for endothelial cell_VWF) defined by our previous study;^41^ and domain 15 cells are also surrounded by myofibroblast (*MYLK*) and follicular B cell (*MS4A1*).

Another interesting feature of stMVC is able to distinguish normal cells and cancer cells, by utilizing the histological features where normal cells and cancer cells have different texture features in terms of size and shape. To clarify this, we designed a tumor suppressor gene- and oncogene-based statistical model to detect normal cells (see “Statistical model for detecting normal cells”). For example, in ovarian cancer, we observed that normal cells with over-expression of suppressor genes (i.e., *TP53* and *BRCA2*)^42^ and lower-expression of oncogenes (i.e., *MYC* and *NME1*)^43^ are mainly enriched in domains 4, 5, 8, and 9, and normal and cancer cells are separated at the UMAP space (Supplementary Fig. 10c–e).

In short, stMVC is more conductive to clarify tissue heterogeneity in terms of detecting normal cells and tumor cells related to cell proliferation and migration in different spatial regions, which has the potential for clinical and prognostic applications.

### stMVC enables us to identify transited cell-states

**Fig. 4:**
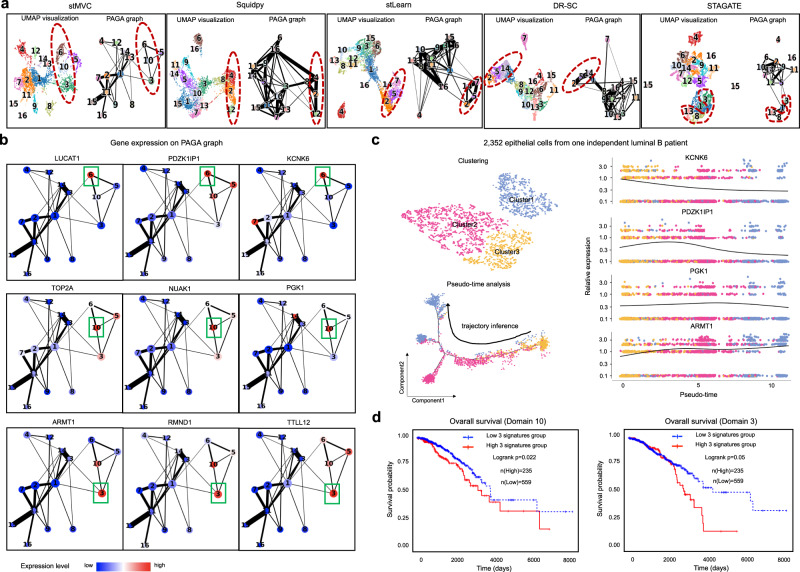
stMVC is able to identify tumor-related cell-states and their transition cell-states from the invasive carcinoma region in the breast cancer sample. **a** UMAP visualization and PAGA graph generated by the low-dimensional features by stMVC, Squidpy, stLearn, DR-SC, and STAGATE, respectively. The predicted clusters for each method are the same as in Fig. 3i. **b** Gene expression levels of nine marker genes for three different cell-states on the PAGA graph by the low-dimensional features of stMVC. **c** Visualization of the clustering and trajectory inference from 2352 epithelial cells of CID4067 (an independent luminal B patient), and pseudo-time-dependent changes in expression levels of *KCNK6*, *PDZK1IP1*, *KGK1*, and *ARMT1*. Each color indicates one cluster. **d** Total survival rate of patients with the high or low expression level of three representative signature genes of domain 10 (i.e., *TOP2A*, *NUAK1*, and *PGK1*, $p=0.022$) and domain 3 (i.e., *ARMT1*, *RMND1*, and *TTLL12*, $p=0.05$) in the RNA-seq data of breast cancer from TCGA database. The logrank test was used for the survival analysis. These breast cancer patients were classified into two groups based on their expression (high $> 30\%$ value, low $< 70\%$ value) for comparison of survival. Note that 794 samples with Luminal A, Luminal B, and Her2 subtypes by PAM50 were used in our survival analysis. Source data are provided as a Source Data file.

To further demonstrate the application power of stMVC, we analyzed the result of breast cancer by stMVC to elucidate its intratumoral heterogeneity and infer possible trajectories in cancer development. The elevated expression of human epidermal growth factor receptor (HER)2 and estrogen receptor (ER) throughout the tumor regions and minimal expression of progesterone receptor (PR) for the data from stMVC, which is in line with the clinical report of Luminal B (Supplementary Fig. 11). More interestingly, we observed that stMVC (i) detects four domains in the ER^+^ invasive carcinoma region outlined with red color, and the detected domains 10 and 3 are very different from DR-SC, Squidpy, stLearn, BayesSpace, and Giotto; (ii) predicts a possible trajectory between three domains, i.e., domain 6 → domain 10 → domain 3, where the trajectory is also detected by DR-SC, Squidpy and stLearn, and the inferred trajectories by those four computational methods can be further validated by trajectory algorithm PAGA^44^; and (iii) similar to stMVC, STAGATE can detect domain 10 cells, however, the trajectory detected by it is very distinct from that by other methods, i.e., there is no direct trajectory between domains 10 and 3 (Figs. 3i and 4a and Supplementary Figs. 8b and 12a).

We validated our predictions by the following several independent ways. Specifically, we noted that (i) four domains have different functions, i.e., domain 6 cells show the high expression levels of genes such as *LUCAT1* and *PDZK1IP1* for regulating breast cancer cell stemness^45,46^; domain 10 cells show overexpression of *NUAK1* and *TOP2A* correlated with the differentiation and stage or grade of the carcinoma^47,48^; domain 3 cells expressing *ARMT1* and *RMND1* within breast cancer susceptibility locus affect cell proliferation^49^; and domain 5 cells with overexpression of *MMP1* and *MMP11* mediate cell invasion and apoptotic process^50^ (Fig. 4b and Supplementary Figs. 12a and 13); (ii) by re-analyzing an independent scRNA-seq data of 24,489 epithelial cells from 20 breast cancer samples, the higher expression of *ARMT1* and *RMND1* is in scRNA-seq data from ER^+^ patients, compared with those from ER^−^ patients (Supplementary Fig. 12b), which is consistent with the previous conclusion: ER, *ARMT1*, and *RMND1* are co-expressed^51^, as well as domains 10 and 3 cells existing in breast cancer; (iii) there is a trajectory between three clusters identified from independent scRNA-seq data of 2352 epithelial cells of CID4067 (a representative luminal B patient, Supplementary Fig. 12b) by Monocle 2^52^, and with the estimated pseudo-time, the expression of *KCNK6* and *PDZK1IP1* for domain 6 decreases, *ARMT1* for domains 10 and 3 increases, and especially *PGK1* for domain 10 is highly expressed at the middle state, supporting our trajectory inference between domains 6, 10, and 3 (Fig. 4c); and (iv) the average expression of three representative signature genes for domain 10 (i.e., *TOP2A*, *NUAK1*, and *PGK1*, $p=0.022$) and 3 (i.e., *ARMT1*, *RMND1*, and *TTLL12*, $p=0.05$) is significantly correlated with shorter overall survival, which was evaluated by independent breast cancer patients from TCGA database (Fig. 4d). Hence, these results indicate that there is possible cell development from cancer stem cells to malignant cells.

Taken together, stMVC can identify cancer-related cell-states and transited cell-states while supporting clinical and prognostic cancer applications from SRT data.

### stMVC improves the results for detecting layer-specific inhibitory neurons in mouse brain

**Fig. 5:**
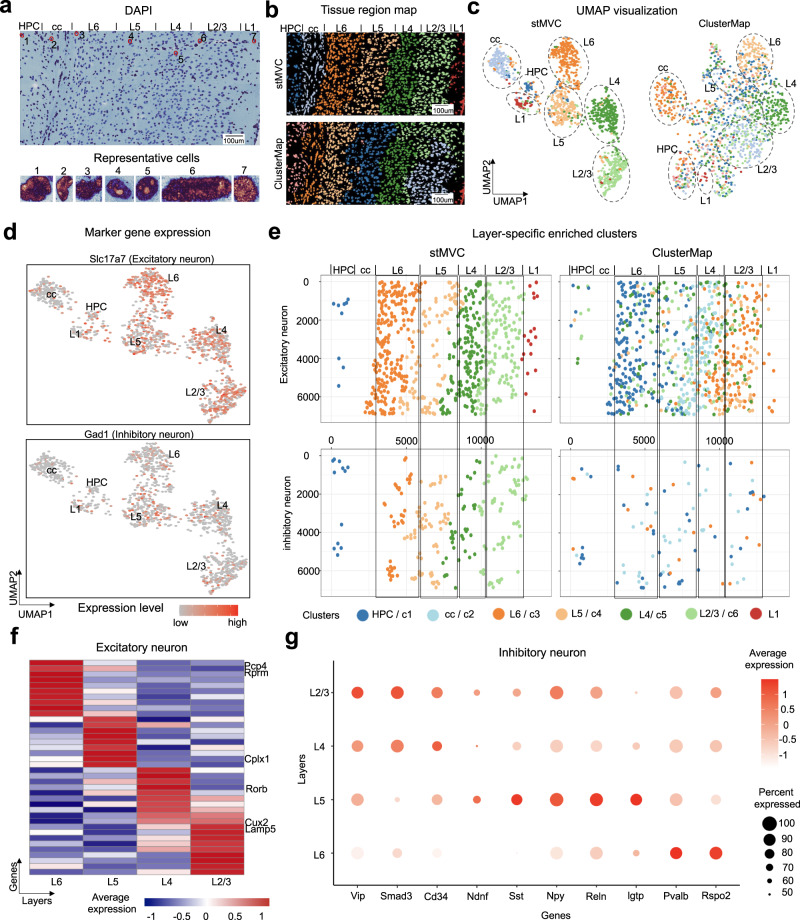
stMVC enables the identification of layer-specific excitatory and inhibitory neurons in the mouse primary visual cortex (V1) dataset. **a** Raw DAPI image of the V1 tissue annotated with seven functionally distinct layers (up panel). Seven representative cells from different layers (bottom panel). **b** Tissue region map predicted by stMVC and ClusterMap. **c** UMAP visualization of the latent features by stMVC and ClusterMap. The layer annotation and color for each cell are the same with (**b**). **d** UMAP visualization (by stMVC) of the marker genes *Slc17a7* and *Gad1* for excitatory and inhibitory neurons, respectively. **e** The spatial map of excitatory and inhibitory neurons predicted by stMVC and ClusterMap, respectively. Each color indicates one cluster. **f** Heatmap of the average gene expression of signature genes for four domains of excitatory neurons in L2-6 by stMVC. Rows and columns indicate genes and different layers, respectively. **g** Dot plot showing the expression levels of marker genes for different subtypes of inhibitory neurons in L2-6 by stMVC. Source data are provided as a Source Data file.

In addition to cancer data from the Visium platform, we further demonstrated stMVC on the mouse primary visual cortex (V1) 1020-gene dataset from the STARmap platform^3^, to detect cell-states from functionally distinct layers. We downloaded RNA clusters per cell predicted by ClusterMap, annotated seven distinct layers (or regions) from raw fluorescence data based on our segmentation strategy (Fig. 5a, b), randomly selected 70% of manual regions to supervise the training of the stMVC, and visualized 10-dimensional features from stMVC by mapping them into two-dimensional UMAP. Subsequently, for each of the seven layers, we predicted cell clusters by the Louvain algorithm to identify excitatory and inhibitory neurons by checking the distribution of their classical marker genes^53^ (Supplementary Fig. 14). For comparison, we also applied ClusterMap for spatial clustering and visualization.

We found that (i) each cortex layer is assigned almost by different feature embeddings from stMVC, compared to ClusterMap, and the excitatory (*Slc17a7*) and inhibitory (*Gad1*) neurons are distributed in the L2/3, L4, L5, and L6 canonical layers (Fig. 5c, d); (ii) stMVC and ClusterMap can accurately detect the layer-specific distribution of excitatory neurons in the L2-6 layers, i.e., layer-specific genes such as *Nov*, *Rorb*, *Cplx1*, and *Pcp4* are highly expressed in L2/3, L4, L5, and L6, respectively^3^ (Fig. 5e, f). However, stMVC is also able to detect a layer-specific pattern of inhibitory neurons, i.e., Sst^+^ and Pvalb^+^ neurons tend to be enriched in L5-6 layers while Vip^+^ neuron tends to be enriched in L2-4 layers (Fig. 5e, g), which is consistent with the previous study^53^. The application of the brain dataset further illustrated that stMVC is able to find cell-states related to the distinct functional regions.

## Discussion

In this work, we proposed stMVC for analyzing SRT data to disentangle the heterogeneity of tissue, especially for the tumor, which integrates four-layer profiles: histological image data, spatial location, gene expression, and region segmentation (i.e., global position) indicating biological contexts, by attention-based multi-view graph collaborative learning. Such tumor position information in the tissue structure used by stMVC can help us to elucidate intratumoral heterogeneity. Different from previous methods that integrate histology and spatial location data by a user-defined weight, for example, SpaGCN manually adjusts the weight of histology in gene expression smoothing, stMVC adopts an attention-based strategy to automatically learn weights of different views for robust representations. Besides, our feature extraction framework from the ResNet-50 model trained for histological image data by data augmentations and contrastive learning did help stMVC to learn more efficient visual features, compared with those from the pre-trained ResNet-50 mode by ImageNet, which was used by stLearn (see the comparison results between SGATE-HSG and SGATE-HSG-N detailed at Fig. 2b–e and Supplementary Figs. 1–6 and 8). The evaluations on two real cancer datasets demonstrated the advantages of stMVC described above, which are able to detect cell-states related to cell stemness, migration, and metastasis distributed in different spatial domains, providing biological insights into tumor heterogeneity. In particular, for the breast cancer dataset, we demonstrated potential clinical and prognostic applications from SRT data, by identifying cancer-related cell-states and also transition cell-states missed by competing methods, which were further validated by clinical data.

By comparing stMVC with the three SGATE-based single-view models, we found that stMVC has a better performance in terms of clustering, inference of trajectory, and denoising, which is mainly attributed to the collaborative learning of multi-view graphs. Besides, we observed that SGATE-based spatial-location-graph model performs better than SGATE-based histological-similarity-graph model, however, SGATE-based histological-similarity-graph model is able to capture some rich boundary information as a complementary to SGATE-based spatial-location-graph model. Hence, we believed that the perspective of modeling SRT data by multi-view graphs enables a better understanding of the tissue heterogeneity, compared with that by single-view graph.

In addition, by comparing with the mean-based strategy stMVC-M, we noted that stMVC achieves better and comparable performance. Specifically, (i) regarding histology without rich texture information, such as DLPFC and ovarian cancer samples, stMVC-M is more vulnerable to the noise signals from the histological visual features while stMVC is easier to capture the finer structure by automatically learning the weight of each of multiple graphs; (ii) regarding histology with rich tissue anatomical structure, like breast cancer sample, both models have a similar result. Hence, we also implemented the mean-based strategy in stMVC model as an option for users to select.

As of now, developing models for integrating SRT data from multiple samples are facing several challenges, for example, the batch effect of gene expression data from multiple sources, as well as their sparsity and noise; constructing the association between spots from different physical metric spaces; and removing the artifacts of histology while creating the relationship between multiple samples^54,55^. However, we believed that (i) the visual feature extraction model by data augmentations and contrastive learning provides a solution to construct the association of spots between different samples; and (ii) the multi-view graph collaborative learning model can provide a novel perspective to integrate multiple SRT datasets by combining multi-layer profiles data. Besides, stMVC is easily scalable to process spatially resolved chromatin accessibility (ATAC-seq) or proteomics data^56,57^, by replacing the feature matrix from gene expression data with that from ATAC-seq or proteomics data. Furthermore, with the advance of spatial multi-omics technology^58^, stMVC can be easily adjusted to adapt by either adding more graphs created by different omics data or substituting the feature matrix from single-omics data with that from multi-omics data fusion^59,60^. Similar to single-view GAT models such as STAGATE, stMVC can be applied to analyze the SRT data of other sequencing-based technologies such as Slide-seq^61^ and Slide-seqV2^62^. Additionally, by exploiting spatiotemporal information derived from stMVC, we can calculate spatial (dynamic) network biomarkers^63–66^ or metabolic states^67,68^ for accurately and reliably quantifying biological systems and further predicting their complex dynamics/behaviors.

We benchmarked the running time of stMVC on the simulated datasets by subsampling spots from the human DLPFC datasets. We observed that stMVC is fast, and takes 38 min to process the SRT dataset with 20 K spots. In particular, the running time is approximately linearly related to the number of input spots (Supplementary Fig. 15), which is considered as an advantage of stMVC for processing a bigger dataset. In our future work, we will further improve the scalability of stMVC, e.g., by introducing a subgraph sampling training strategy.

Some limitations still are found in stMVC: (i) compared with the visual features extraction framework from the ResNet-50 model pre-trained by the ImageNet, the preprocessed step for training SimCLR needs more computational resources and times; and (ii) region segmentation for tumor position is manually annotated based on the staining density of antibodies. With the exploration of deep learning frameworks of generalizable segmentation tools^69,70^, we will further investigate creating a more efficient stMVC model with a more automatic architecture in the future study.

## Methods

### stMVC model

stMVC is a multi-view graph collaborative learning model, which integrates four-layer profiles: gene expression ($X\in {R}^{m\times n}$), spatial location ($S=\left({s}_{1},\ldots,{s}_{n}\right)\in {R}^{n\times 2},{s}_{i}=({s}_{{ix}},{s}_{{iy}})$), histological image data ($I=\left({i}_{1},\ldots,{i}_{n}\right)$), and manual region segmentation ($Y={({y}_{1},\ldots,{y}_{n})}^{T}\in {R}^{n\times 1},{y}_{i}\in \{1,\ldots,K\}$), where $m$, $n$, and $K$ are the number of genes, spots, and spatial domains (manual segmentation), to elucidate tumor heterogeneity (Fig. 1a–d). Specifically, we (1) learned visual features (${h}_{i}\in {R}^{2048\times 1}$) for each spot (or cell) image (${i}_{i}$) by feature extraction network of the ResNet-50 model that was trained by SimCLR framework^71^, and then constructed HSG based on the similarity of the learned visual features (${h}_{i}$) (Fig. 1b); (2) separately learned view-specific representations (${P}^{m}=\big({{p}_{1}}^{m}$,…, ${{p}_{n}}^{m}\big)\in {R}^{d\times n}$, where $d$ is the number of latent features, $m\in \left\{{{{{\mathrm{1,2}}}}}\right\}$ indicating $m$th graph) by SGATE for each of two-view graphs: HSG and SLG, as well as low-dimensional feature of $X$ by an autoencoder as a feature matrix ($Z\in {R}^{b\times n}$, where the default value of $b$ is 50, indicating the dimension of latent features), and meanwhile automatically learned the weights of two graphs for robust representations ($R=({r}_{1},..,{r}_{n})\in {R}^{d\times n}$) by attention mechanism, in a semi-supervision manner from region segmentation (Fig. 1c).

### Learning visual features by contrastive learning to construct HSG

To remove the noise from histology staining and extract morphological information from histological image data, we adopted SimCLR model to efficiently learn visual features for each spot by maximizing agreement between differently augmented views of the same spot image via a contrastive loss in the latent space (Fig. 1b). For a spot ${v}_{i}$ from the Visium platform, the physical location of which in the tissue slice is represented by $({s}_{{ix}},{s}_{{iy}})$, a square containing $40\times 40$ pixels centered on $({s}_{{ix}},{s}_{{iy}})$ is regarded as its image ${i}_{i}$; and for a cell ${v}_{i}$ from the STARmap platform, a minimum enclosing bounding rectangle for a cell determined by its RNA locations is treated as its image ${i}_{i}$. The specific model is described as follows:

1. For each image ${i}_{i}$, we applied a stochastic data augmentation module to randomly transform it into two correlated views for the same image ${i}_{i}$, denoted as ${{i}_{m}}^{{\prime} }$ and ${{i}_{n}}^{{\prime} }$, and considered these two views as a positive pair. In this work, we sequentially apply two simple augmentations: random cropping followed by resizing back to the original size; and random color distortions including randomly changing the brightness, contrast, saturation, and hue of an image^71^.
2. We adopted the ResNet-50 framework^24^ as a base encoder $f(\bullet )$ to extract features (${h}_{m}$ and ${h}_{n}$) from two augmented views (${{i}_{m}}^{{\prime} }$ and ${{i}_{n}}^{{\prime} }$), where ${h}_{m},{h}_{n}\in {R}^{2048\times 1}$ is the output after average pooling layer.
3. We used a Multilayer Perceptron (MLP) with one hidden layer to obtain latent features ${l}_{m}=g\left({h}_{m}\right)={W}^{\left(2\right)}\sigma \left({W}^{\left(1\right)}{h}_{m}\right)$ where $\sigma$ is a ‘relu’ function, the first and second layers are set as 512 and 128, respectively, and ${l}_{m}$ is used to define contrastive loss.
4. Given a set $\left\{{{i}_{k}}^{{\prime} }\right\}$ including a positive pair of examples ${{i}_{m}}^{{\prime} }$ and ${{i}_{n}}^{{\prime} }$, the contrastive prediction task aims to identify ${{i}_{n}}^{{\prime} }$ in ${\left\{{{i}_{k}}^{{\prime} }\right\}}_{k\ne m}$ for a given ${{i}_{m}}^{{\prime} }$. The loss function for a positive pair of samples $({{i}_{m}}^{{\prime} },{{i}_{n}}^{{\prime} })$ is defined as:

1 $${l}_{i,j}=-\,\log \frac{\exp ({{{{{\rm{sim}}}}}}({l}_{m},{l}_{n})/\tau )}{{\sum }_{k=1}^{2N}{{\mathbb{I}}}_{[k\ne m]}\exp ({{{{{\rm{sim}}}}}}({l}_{m},{l}_{n})/\tau )}$$

where ${{{\mbox{sim}}}}\left({l}_{m},{l}_{n}\right)=\frac{{{l}_{m}}^{{{{\rm T}}}}{l}_{n}}{||{l}_{m}||{||}{l}_{n}{||}}$, ${{\mathbb{I}}}_{\left[k\ne m\right]}\in \{{{{{\mathrm{0,1}}}}}\}$ is an indicator function evaluating to 1 if $k\ne m$, $\tau$ denotes a temperature parameter, and $N$ indicates batch size.

For each SRT data, we randomly selected 85% of the spots as the training sets to train the SimCLR model, and the remaining 15% of the spots as the testing set to test it. Adam optimizer with both a ${1e}^{-6}$ weight decay and a ${1e}^{-4}$ learning rate is used to minimize the above loss function. We trained the SimCLR model until 500 iterations, and then calculated the histological similarity between any two spots by calculating the Cosine similarity between learned features ${h}_{u}$ and ${h}_{v}$ from spots $u$ and $v$ as follows: ${\cos }\left({h}_{u},{h}_{v}\right)=\frac{{h}_{u}\bullet {h}_{v}}{\left|\left|{h}_{u}\right|\right|{||}{h}_{v}{||}}$. A Six-nearest neighbor graph for each spot is kept for constructing the HSG ${G}^{1}=(V,{E}^{1})$, where each vertex $v\in V$ indicates a spot, and every two vertices in $V$ are connected by an edge $e\in {E}^{1}$. As a comparison, we used the same strategy to construct naïve HSG ${{G}^{1}}^{{\prime} }=(V,{{E}^{1}}^{{\prime} })$ by the feature extracted by feature extraction framework of ResNet-50 pre-trained by ImageNet to validate the effectiveness of our trained visual feature extraction model.

### Learning robust representations by multiple view graph collaborative learning

We used SGATEs for learning view-specific representations in stMVC, where a SGATE for each view was trained under weak supervision of the region segmentation to capture its efficient low-dimensional manifold structure by integrating gene expression with either HSG (${G}^{1}$) or SLG, (${G}^{2}=(V,{E}^{2})$) which is constructed by a six-nearest neighbor graph for each spot based on the Euclidean distance of their physical location in the tissue slice^72^, and meanwhile collaboratively integrated them to learn robust representations for each spot (Fig. 1c). In this work, two graphs (${G}^{1}$ and ${G}^{2}$) have the same vertex (spot) set but different edges.

#### stMVC for learning view-specific representations

For each view graph, a SGATE model aims to learn accurate low-dimensional features with the following inputs: an adjacency matrix (${A}^{m}\in {R}^{n\times n}$) representing $m$th graph ${G}^{m}$, where $m\in \left\{{{{{\mathrm{1,2}}}}}\right\}$, cell labels indicated by region segmentation ($Y$), and feature matrix $Z$ learned from gene expression ($X$) by autoencoder-based framework (see Supplementary Methods). A GAT can be built by stacking multiple multi-head graph attention layers^73^. Specifically, each layer is defined as:

2 $${{h}_{i}}^{(l+1)}=\sigma \left(\frac{1}{Q}\mathop{\sum }\limits_{q=1}^{Q}\mathop{\sum}\limits_{j\in {N}_{i}}{{\alpha }_{{ij}}}^{q}{W}^{q}{{h}_{j}}^{l}\right)$$

3 $${{\alpha }_{ij}}^{q}=\frac{\exp ({{{{{\rm{LeakyReLU}}}}}}({({a}^{q})}^{T}[{W}^{q}{{h}_{i}}^{l}||{W}^{q}{{h}_{j}}^{l}]))}{{\sum }_{o\in {N}_{i}}\exp ({{{{{\rm{LeakyReLU}}}}}}({({a}^{q})}^{T}[{W}^{q}{{h}_{i}}^{l}||{W}^{q}{{h}_{o}}^{l}]))}$$

where $Q$ indicates the number of head attention and the default value is two, ${{\alpha }_{{ij}}}^{q}$ is normalized attention coefficients computed by the $q$th attention mechanism (${a}^{q}$), ${W}^{q}$ is the corresponding input linear transformation’s weight matrix, ${N}_{i}$ is the neighborhood of spot (${v}_{i}$) in the graph, ${{h}_{j}}^{l}$ is the input feature of node $j$ of the $l$th layer, and $\parallel$ is the concatenation operation.

The encoder of each GATE model is composed of two layers of GAT, the output dimensions of the first and second layers are set at 25 and 10, 36 and 18, and 32 and 16, for human DLPFC, ovarian, and breast cancer datasets, respectively. The graph embedding for each graph is represented by ${P}^{m}$. The decoder of each GATE model is defined as an inner product between the embedding:

4 $${{A}^{m}}^{{\prime} }={{{{{\rm{sigmoid}}}}}}({P}^{m}{P}^{mT})$$

where ${{A}^{m}}^{{\prime} }$ is the reconstructed adjacency matrix of ${A}^{m}$. The goal of learning each GATE model is to minimize the cross-entropy between the input adjacency matrix ${A}^{m}$ and ${{A}^{m}}^{{\prime} }$:

5 $${L}_{{{{{{\rm{recon}}}}}}{\mbox{-}}{m}}=-\frac{1}{n\times n}\mathop{\sum }\limits_{i=1}^{n}\mathop{\sum }\limits_{j=1}^{n}({{a}_{ij}}^{m}\times \,\log ({{a}_{ij}}^{m{\prime} })+(1-{{a}_{ij}}^{m})\times \,\log (1-{{a}_{ij}}^{m{\prime} }))$$

where ${{a}_{{ij}}}^{m}$ and ${{{a}_{{ij}}}^{m}}^{{\prime} }$ are the elements of the adjacency matrix ${A}^{m}$ and ${{A}^{m}}^{{\prime} }$ in the $i$th row and the $j$th column of $m$th graph, respectively.

To capture biological contexts in the tissue, we further extended the use of the GATE model to do spot class prediction ${{Y}^{m}}^{{\prime} }={{{\mbox{softmax}}}}({{W}_{m}}^{(1)}{P}^{m})$ in a semi-supervision manner from region segmentation, and the loss function of which is summarized as follows:

6 $${L}_{{{{{{\rm{pre}}}}}}{\mbox{-}}{m}}=\frac{1}{S}\mathop{\sum }\limits_{l=1}^{S}\left(-\mathop{\sum }\limits_{i=1}^{K}{y}_{i}\,\log ({{y}_{i}}^{m{\prime} })\right)$$

where $S$ is the number of labeled spots, $K$ is the number of classes, and ${y}_{i}$ and ${{{y}_{i}}^{m}}^{{\prime} }$ are the label vector of spot ${v}_{i}$ from the region segmentation and the prediction of the $m{{{{{\rm{th}}}}}}$ graph, respectively.

Taken together, the loss function of each SGATE model is summarized as:

7 $${L}_{{{{{{{\rm{SGATE}}}}}}}{\mbox{-}}m}={L}_{{{{{{\rm{recon}}}}}}{\mbox{-}}m}+{\beta L}_{{{{{{\rm{pre}}}}}}{\mbox{-}}m}$$

where $\beta$ is a parameter used to control the weight of two loss functions, and the default value is eight.

#### stMVC for multi-view graph collaborative learning

After learning view-specific representations ${{p}_{i}}^{m}$ for a spot (${v}_{i}$) by the $m$th SGATE model, we applied the collaborative learning model to integrate different view graphs for its robust representations (${r}_{i}$). The contribution of each view to ${r}_{i}$ is unavailable, hence, we proposed the attention-based strategy to learn the weight of each graph for the final representations by the following function:

8 $${r}_{i}=\mathop{\sum }\limits_{m=1}^{M}{{\gamma }_{i}}^{m}{{p}_{i}}^{m}$$

where $M$ is the number of views. Inspired by attention-based models emphasizing on capturing more critical information to the current task from abundant information^22,74^, we defined the weight of one view for node ${v}_{i}$ using the following function:

9 $${{\gamma }_{i}}^{m}=\frac{\exp ({a}_{m}\cdot {{p}_{i}}^{C})}{{\sum }_{o=1}^{M}\exp ({a}_{o}\cdot {{p}_{i}}^{C})}$$

where ${{p}_{i}}^{C}\in {R}^{2d\times 1}$ is the concatenation of all view-specific representations of spot ${v}_{i}$, and ${a}_{m}\in {R}^{2d\times 1}$ is feature vector of the $m$th view, describing what kinds of spots will consider the $m$th view as informative. If ${{p}_{i}}^{C}$ and ${a}_{m}$ have a large dot product, meaning spot ${v}_{i}$ believes that the $m$th view is an informative view, and vice versa.

To collaboratively integrate different views into the same semantic space^60^, we further leveraged robust representations to fine-tune the learning of each view graph by transferring knowledge from robust representations to each view-specific representations, as well as optimizing the parameters of ${\left\{{a}_{m}\right\}}_{m=1}^{M}$ in semi-supervised manner through a MLP with one hidden layer to do spot class prediction ${{Y}^{{CL}}}^{{\prime} }={{{\mbox{softmax}}}}({W}^{\left(2\right)}\sigma ({W}^{\left(1\right)}R))$. The corresponding loss functions are defined as follows:

10 $${L}_{{{{{{\rm{transfer}}}}}}}=\mathop{\sum }\limits_{i=1}^{n}\mathop{\sum }\limits_{m=1}^{M}{{\gamma }_{i}}^{m}{({r}_{i}-{{p}_{i}}^{m})}^{2}$$

11 $${L}_{{{{{{\rm{pre}}}}}}{\mbox{-}}{{{{{\rm{CL}}}}}}}=\frac{1}{S}\mathop{\sum }\limits_{l=1}^{S}\left(-\mathop{\sum }\limits_{i=1}^{K}{y}_{i}\,\log ({{y}_{i}}^{CL{\prime} })\right)$$

In summary, the total loss function for robust representations by collaboratively integrating multiple views is summarized as follows:

12 $${L}_{{{{{{\rm{MVC}}}}}}}=\mathop{\sum }\limits_{m=1}^{M}{L}_{{{{{{\rm{recon}}}}}}{\mbox{-}}m}+{\beta }^{{\prime} }\mathop{\sum }\limits_{m=1}^{M}{L}_{{{{{{\rm{pre}}}}}}{\mbox{-}}m}+\delta {L}_{{{{{{\rm{transfer}}}}}}}+\varphi {L}_{{{{{{\rm{pre}}}}}}{\mbox{-}}{{{{{\rm{CL}}}}}}}$$

where ${\beta }^{{\prime} }$, $\delta$ and $\varphi$ are used to control the weights of these regularization terms, and the default values of them are 10, 0.0005, and 100, respectively.

Overall, the objective function of our stMVC model can be effectively optimized with the following iterations, specifically, for each iteration, we (i) optimized the parameters of each SGATE model to learn the view-specific representations based on Eq. (7), as well as Eq. (10) when the robust representations ($R$) has been inferred by the Eq. (8), and followed by inferring $R$ via Eq. (8); and (ii) optimized the parameters of all SGATE models and the parameter vectors of all graphs based on Eq. (12), and then inferred $R$ based on the optimized stMVC model. We trained the stMVC model until convergence, and then applied $R$ for the spatial clustering, visualization, and denoising (Fig. 1d).

### Datasets and preprocessing

#### SRT data

In our study, human DLPFC, ovarian, and breast cancer datasets with paired gene expression and histology were publicly available from the 10X Genomics website. Specifically, (i) DLPFC dataset with 12 slices each is manually annotated with the six cortical layers and WM by the previous study, where the number of spots ranging from 3460 to 4789 with a median of 3844^26^. Besides, for each slice, we randomly selected manual annotation with the proportion ranging from 0.1 to 0.9 by 0.1, thus generating nine manual annotation sets, which were used to check if or not stMVC can capture the inner structure of tissue by few labels; (ii) ovarian cancer sample with matching gene expression (3493 spots) and immunofluorescence staining with an anti-human CD45 antibody and DAPI; and (iii) breast cancer sample with paired gene expression data (4727 spots) and immunofluorescence staining with an anti-human CD3 antibody and DAPI. In addition, a mouse primary visual cortex (V1) 1020-gene sample with matching gene expression (1365 cells predicted by ClusterMap) and raw immunofluorescence staining with DAPI was available from STARmap platform^3^.

#### scRNA-seq and bulk RNA-seq data

To validate our predictions for the sample of ovarian cancer, we extracted 4081 epithelial cells from one ovarian cancer patient from a previous study^30^. Besides, 24,489 epithelial cells were retrieved from 20 breast cancer patients from previous research, including five luminal B, three Luminal A, three HER2^+^, seven triple-negative breast cancers (TNBCs), one HER2^+^ and ER^+^, and one normal^75^, to support our findings, and the subtypes are determined by their clinical reports and predictions. Furthermore, the bulk RNA-seq and clinical data of breast cancer in TCGA database were downloaded from the Xena platform for survival analysis^76^, and the PAM50 subtype classifications of breast cancer of TCGA were downloaded from previous research^77^.

#### Preprocessing

The top 2000 highly variable genes by the ‘vst’ method of Seurat^78^ for each gene expression data from the Visium platform, and all 1020 genes of the mouse primary visual cortex dataset from the STARmap platform, were used to comprehensively compare each computational method. Besides, to efficiently capture the information within gene expression data, we mapped each data into 50-dimensional latent features based on our designed autoencoder-based framework (see Supplementary Methods), and took them as the input of stMVC.

### Manual region segmentation

We designed a strategy to define biological contexts in each tumor tissue based on the following assumptions: (1) the colors of the antibodies on the immunofluorescence staining of the tumor histology can roughly define the tumor region; (2) cells belonging to the same cell type but are separated by other cells such as immune or other mesenchymal cells may have different cell-states; and (3) the majority of cells in the tumor region are tumors while a few are normal epithelial and infiltrating stromal or immune cells, and the tumor purity was larger than 70%, which was estimated by over 9300 tumors of 21 cancer types from the TCGA database^79^ (Supplementary Fig. 16). After annotating different regions in the histology image by labelme software^80^, we further applied the OpenCV package^81^ to determine the region (or context) to which each spot (${v}_{i}$) belongs, by calculating the proportion between area of the intersection between a square (${{{\mbox{IR}}}}_{i}$) containing $40\times 40$ pixels centered on $({s}_{{ix}},{s}_{{iy}})$ and a region (${R}_{j}$), compared to the square. The function is defined as follows:

13 $${{{{{\rm{Proportion}}}}}}=\frac{{{{{{\rm{Intersection}}}}}}({{{{{{\rm{IR}}}}}}}_{i},{R}_{j})}{I{R}_{i}}$$

By setting ${{\mbox{Proportion}}}\ge 0.5$, for the ovarian cancer sample, 1658 spots were separately classified into 17 different regions in the tumor, and the remaining 1835 spots in the non-tumor region were treated as the 18th region (Supplementary Fig. 6a); and for the breast cancer sample, 2091 spots were classified into 15 different regions in the tumor, and the remaining 2636 spots were regarded as the 16th region (Supplementary Fig. 8a).

In addition, we further extended the protocol to process imaging-based SRT data. Specifically, we predicted RNA clusters for each cell by ClusterMap; for each cell ${v}_{i}$, treated a minimum enclosing bounding rectangle containing the cell determined by its RNA’s physical locations as ${{{\mbox{IR}}}}_{i}$; and adopted Eq. (7) to predict the region to which the cell belongs. By computing, for the mouse V1 sample, 1365 cells were classified into seven regions (Fig. 5b).

### Evaluation of denoising of gene expression data

We adopted a GI-based measure^60^ to quantify the quality of denoised gene expression data by estimating a degree of inequality in the distribution of known layer-specific gene expression levels. Specifically, for each domain of the ground truth, the average expression of each marker gene was calculated; and then the GI for each gene calculated by the ‘gini’ function from reldist package was used to evaluate the specific level of the marker gene^82^. The higher the GI score, the better the denoised data. The marker genes used in DLPFC dataset were downloaded from the previous study^26^ (Supplementary Table 2).

### Statistical model for detecting normal cells

To clarify that stMVC is able to distinguish normal cells from cancer cells by integrating the histological features, we further designed a statistical-based measure to detect normal cells from SRT data. Specifically, we reasoned that (1) normal cells tend to be with a higher expression of tumor suppressor genes while cancer cells tend to have a higher expression of oncogenes; and (2) the expression levels between the tumor suppressor genes and the oncogenes are different or not correlated, which were calculated by Fisher’s exact test (see Supplementary Methods). Hence, cells with a higher expression of suppressor genes but a lower expression of oncogenes were regarded as the normal cells, noting that the expressions of suppressor genes and the oncogenes are not correlated (*p*-value > 0.05).

### Clustering and visualization

After applying stMVC in analyzing SRT data, we learned the accurate low-dimensional representations ($R$) representing the relationship between any two spots. To further clarify tissue heterogeneity, given $R$ as the input, we adopted ‘FindNeighbors’ and ‘FindClusters’ function with default parameters from the Seurat package to determine $k$-nearest neighbors (KNNs) for each spot, construct the shared nearest neighbor graph, predict the cell clusters by the Louvain algorithm, and each cluster is considered as a spatial domain.

We utilized the UMAP algorithm to map the low-dimensional features from each computational method to two-dimension, visualized the distance of cell embeddings between different cell populations by ‘Dimplot’ function, and visualized the clusterings and gene expression patterns at the spatial level by ‘SpatialDimPlot’ and ‘SpatialFeaturePlot’ function, respectively.

### Evaluation of the clustering

We adopted two different metrics to evaluate the clustering by calculating the similarity of features between spots within the predicted cell clusters. Specifically, ROGUE^28^, an entropy-based statistic to quantify the homogeneity of identified cell clusters based on transcriptome similarity between spots within each cluster, and the higher the value, the better the clustering; silhouette width^29^, a measure of how similar a spot is to its predicted cluster compared to other clusters, and the higher the value, the spot well belongs to its cluster, which is calculated as follows:

14 $${{{{{\rm{SW}}}}}}(i)=\frac{c(i)-d(i)}{{{\max }}\,\{c(i),d(i)\}}$$

where $d(i)$, and $c\left(i\right)$ indicate the average Euclidean distance of the learned low-dimensional joint features between a spot ($i$) and other spots in the same cluster, and the spot ($i$) to all spots in the nearest cluster where $i$ does not belongs, respectively. The average of silhouette width of all spots as the final metrics (ASW) to evaluate clustering performance.

### Identification of SVGs

We constructed KNN graph for each spot based on the learned low-dimensional representations ($R$), and adopted the KNN-smoothing algorithm to aggregate information from 15 nearest spots for each spot to denoise the gene expression data. Then, we identified SVGs from 2000 highly variable genes among different spatial domains from the stMVC model by ‘FindAllMarkers’ from Seurat package.

### Reporting summary

Further information on research design is available in the Nature Research Reporting Summary linked to this article.

## Supplementary information

Supplementary Information

Reporting Summary

## Data Availability

The raw count matrix, histological image, and spatial location data for both human ovarian and breast cancer samples are publicly available at the 10X Genomics Website (https://support.10xgenomics.com/spatial-gene-expression/datasets). The raw count matrix, image, and spatial location data for 12 slices of human DLPFC dataset are available from the package spatialLIBD (http://spatial.libd.org/spatialLIBD/)^26^. The DAPI image and RNA clusters per cell for mouse primary visual cortex 1020-gene sample are publicly available from GitHub link of ClusterMap (https://github.com/wanglab-broad/ClusterMap). The additional scRNA-seq data of 20 human breast cancers and one human ovarian cancer are publicly available from Gene Expression Omnibus database under accession code GSE176078 and EMBL-EBI database under accession code E-MTAB-8859, respectively. The bulk RNA-seq and clinical data from the TCGA database are at the Xena platform (https://xenabrowser.net/datapages/). The functional gene sets are at MSigDB database (https://www.gsea-msigdb.org/gsea/msigdb/). Source data are available at figshare^83^. Source data are provided with this paper.

## Source data

Source data

## Code availability

stMVC is implemented based on python 3.6.12 and R 4.0.0. Other tools and packages used in the data analysis include: numpy 1.19.2, pandas 1.1.5, scipy 1.5.2, scikit-learn 0.23.3, torch 1.6.0, tqdm 4.55.0, scanpy 1.6.0, PIL 9.1.0, seaborn 0.11.2, sklearn 1.0.2, matplotlib 3.5.2, glob2, anndata 0.8.0, argparse 1.1, json 2.0.9, R 4.0.0, Seurat v4, ggplot2 3.3.5, monocle 2.10.1, SpatialDecon 1.6.0, DR-SC 2.9, STAGATE 1.0.1, cv2 (OpenCV) 4.5.5, labelme 5.0.1, ClusterMap 0.0.1, Squidpy 1.2.2, stLearn 0.3.1, BayesSpace 1.1.4, and Giotto 1.0.4. The codes are available at Zenodo https://zenodo.org/record/6052602^84^. The stMVC tool will be maintained and updated at https://github.com/cmzuo11/stMVC.

## References

[1] Chen A (2022). Spatiotemporal transcriptomic atlas of mouse organogenesis using DNA nanoball-patterned arrays. Cell.

[2] Moses L, Pachter L (2022). Museum of spatial transcriptomics. Nat. Methods.

[3] Wang X (2018). Three-dimensional intact-tissue sequencing of single-cell transcriptional states. Science.

[4] Ståhl PL (2016). Visualization and analysis of gene expression in tissue sections by spatial transcriptomics. Science.

[5] Hunter M, Moncada R, Weiss J, Yanai I, White R (2020). Spatial transcriptomics reveals the architecture of the tumor/microenvironment interface. Nat. Commun..

[6] Liao J, Lu X, Shao X, Zhu L, Fan X (2021). Uncovering an organ’s molecular architecture at single-cell resolution by spatially resolved transcriptomics. Trends Biotechnol..

[7] Xiaowei, A. Method of the Year 2020: Spatially resolved transcriptomics. *Nat. Methods* **18**, 1 (2021). 10.1038/s41592-020-01042-x 33408396

[8] Dries R (2021). Advances in spatial transcriptomic data analysis. Genome Res..

[9] Zhao E (2021). Spatial transcriptomics at subspot resolution with BayesSpace. Nat. Biotechnol..

[10] Dries R (2021). Giotto: A toolbox for integrative analysis and visualization of spatial expression data. Genome Biol..

[11] Hu J (2021). SpaGCN: Integrating gene expression, spatial location and histology to identify spatial domains and spatially variable genes by graph convolutional network. Nat. Methods.

[12] Pham, D. et al. stLearn: Integrating spatial location, tissue morphology and gene expression to find cell types, cell-cell interactions and spatial trajectories within undissociated tissues. Preprint at *bioRxiv*10.1101/2020.05.31.125658 (2020).

[13] Palla G (2022). Squidpy: A scalable framework for spatial omics analysis. Nat. Methods.

[14] He Y (2021). ClusterMap for multi-scale clustering analysis of spatial gene expression. Nat. Commun..

[15] Liu W (2022). Joint dimension reduction and clustering analysis of single-cell RNA-seq and spatial transcriptomics data. Nucleic Acids Res..

[16] Yang Y (2022). SC-MEB: Spatial clustering with hidden Markov random field using empirical Bayes. Brief. Bioinform..

[17] Dong K, Zhang S (2022). Deciphering spatial domains from spatially resolved transcriptomics with an adaptive graph attention auto-encoder. Nat. Commun..

[18] Wang J (2021). scGNN is a novel graph neural network framework for single-cell RNA-Seq analyses. Nat. Commun..

[19] Haralick, R. M., Shanmugam, K. & Dinstein, I. H. Textural features for image classification. In *IEEE Transactions on Systems, Man, and Cybernetics* 610–621 (1973).

[20] Velickovic P (2017). Graph attention networks. stat.

[21] Wu Z (2020). A comprehensive survey on graph neural networks. IEEE Trans. Neural Netw. Learn. Syst..

[22] Qu, M. et al. Attention-based collaboration framework for multi-view network representation learning. In *Proceedings of the 2017 ACM on Conference on Information and Knowledge Management* 1767–1776 (2017).

[23] Gurcan MN (2009). Histopathological image analysis: A review. IEEE Rev. Biomed. Eng..

[24] He, K., Zhang, X., Ren, S. & Sun, J. Deep residual learning for image recognition. In *Proceedings of the IEEE Conference on Computer Vision and Pattern Recognition* 770–778 (2016).

[25] Deng, J. et al. Imagenet: A large-scale hierarchical image database. In *2009 IEEE Conference on Computer Vision and Pattern Recognition* 248–255 (2009).

[26] Maynard KR (2021). Transcriptome-scale spatial gene expression in the human dorsolateral prefrontal cortex. Nat. Neurosci..

[27] Nadarajah B, Parnavelas JG (2002). Modes of neuronal migration in the developing cerebral cortex. Nat. Rev. Neurosci..

[28] Liu B (2020). An entropy-based metric for assessing the purity of single cell populations. Nat. Commun..

[29] Rousseeuw PJ (1987). Silhouettes: A graphical aid to the interpretation and validation of cluster analysis. J. Comput. Appl. Math..

[30] Nelson L (2020). A living biobank of ovarian cancer ex vivo models reveals profound mitotic heterogeneity. Nat. Commun..

[31] Danaher P (2022). Advances in mixed cell deconvolution enable quantification of cell types in spatial transcriptomic data. Nat. Commun..

[32] Kulbe H (2007). The inflammatory cytokine tumor necrosis factor-α generates an autocrine tumor-promoting network in epithelial ovarian cancer cells. Cancer Res..

[33] Xia Y, Shen S, Verma IM (2014). NF-κB, an active player in human cancers. Cancer Immunol. Res..

[34] Petrova V, Annicchiarico-Petruzzelli M, Melino G, Amelio I (2018). The hypoxic tumour microenvironment. Oncogenesis.

[35] Nguyen T, Nioi P, Pickett CB (2009). The Nrf2-antioxidant response element signaling pathway and its activation by oxidative stress. J. Biol. Chem..

[36] Al-Alem L, Curry TE (2015). Ovarian cancer: Involvement of the matrix metalloproteinases. Reproduction.

[37] Steitz AM (2020). Tumor-associated macrophages promote ovarian cancer cell migration by secreting transforming growth factor beta induced (TGFBI) and tenascin C. Cell Death Dis..

[38] Guo X, Ding X (2018). Dioscin suppresses the viability of ovarian cancer cells by regulating the VEGFR2 and PI3K/AKT/MAPK signaling pathways. Oncol. Lett..

[39] Zhang Y (2011). Ovarian cancer-associated fibroblasts contribute to epithelial ovarian carcinoma metastasis by promoting angiogenesis, lymphangiogenesis and tumor cell invasion. Cancer Lett..

[40] Wang H (2014). NEDD9 overexpression is associated with the progression of and an unfavorable prognosis in epithelial ovarian cancer. Hum. Pathol..

[41] Zhang Y (2021). Single-cell RNA-sequencing atlas reveals an MDK-dependent immunosuppressive environment in ErbB pathway-mutated gallbladder cancer. J. Hepatol..

[42] Aunoble B, Sanches R, Didier E, Bignon Y (2000). Major oncogenes and tumor suppressor genes involved in epithelial ovarian cancer. Int. J. Oncol..

[43] Youn BS (2008). NM23 as a prognostic biomarker in ovarian serous carcinoma. Mod. Pathol..

[44] Wolf FA (2019). PAGA: Graph abstraction reconciles clustering with trajectory inference through a topology preserving map of single cells. Genome Biol..

[45] Zheng A (2019). Long non-coding RNA LUCAT1/miR-5582-3p/TCF7L2 axis regulates breast cancer stemness via Wnt/β-catenin pathway. J. Exp. Clin. Cancer Res..

[46] Garcia-Heredia JM, Lucena-Cacace A, Verdugo-Sivianes EM, Pérez M, Carnero A (2017). The cargo protein MAP17 (PDZK1IP1) regulates the cancer stem cell pool activating the Notch pathway by abducting NUMB. Clin. Cancer Res..

[47] Chang X-Z, Yu J, Liu H-Y, Dong R-H, Cao X-C (2012). ARK5 is associated with the invasive and metastatic potential of human breast cancer cells. J. Cancer Res. Clin. Oncol..

[48] Rody A (2009). Gene expression of topoisomerase II alpha (TOP2A) by microarray analysis is highly prognostic in estrogen receptor (ER) positive breast cancer. Breast Cancer Res. Treat..

[49] Yamamoto-Ibusuki M (2015). C6ORF97-ESR1 breast cancer susceptibility locus: Influence on progression and survival in breast cancer patients. Eur. J. Hum. Genet..

[50] Gobin E (2019). A pan-cancer perspective of matrix metalloproteases (MMP) gene expression profile and their diagnostic/prognostic potential. BMC Cancer.

[51] Dunbier AK (2011). ESR1 is co-expressed with closely adjacent uncharacterised genes spanning a breast cancer susceptibility locus at 6q25. 1. PLoS Genet..

[52] Trapnell C (2014). The dynamics and regulators of cell fate decisions are revealed by pseudotemporal ordering of single cells. Nat. Biotechnol..

[53] Tasic B (2016). Adult mouse cortical cell taxonomy revealed by single cell transcriptomics. Nat. Neurosci..

[54] Chatterjee S (2014). Artefacts in histopathology. J. Oral. Maxillofac. Pathol.: JOMFP.

[55] Hu J (2021). Statistical and machine learning methods for spatially resolved transcriptomics with histology. Comput. Struct. Biotechnol. J..

[56] Li R, Zhou S (2021). Spatially resolved proteomics identify biomarkers from endometrial sentinel lymph nodes. Cell Rep. Med..

[57] Deng, Y. et al. Spatial profiling of chromatin accessibility in mouse and human tissues. *Nature* **609**, 1–9 (2022). 10.1038/s41586-022-05094-1 PMC9452302 35978191

[58] Liu Y (2020). High-spatial-resolution multi-omics sequencing via deterministic barcoding in tissue. Cell.

[59] Zuo C, Chen L (2021). Deep-joint-learning analysis model of single cell transcriptome and open chromatin accessibility data. Brief. Bioinform..

[60] Zuo C, Dai H, Chen L (2021). Deep cross-omics cycle attention model for joint analysis of single-cell multi-omics data. Bioinformatics.

[61] Rodriques SG (2019). Slide-seq: A scalable technology for measuring genome-wide expression at high spatial resolution. Science.

[62] Stickels RR (2021). Highly sensitive spatial transcriptomics at near-cellular resolution with Slide-seqV2. Nat. Biotechnol..

[63] Chen L, Liu R, Liu Z-P, Li M, Aihara K (2012). Detecting early-warning signals for sudden deterioration of complex diseases by dynamical network biomarkers. Sci. Rep..

[64] Liu X, Wang Y, Ji H, Aihara K, Chen L (2016). Personalized characterization of diseases using sample-specific networks. Nucleic Acids Res..

[65] Liu X (2019). Detection for disease tipping points by landscape dynamic network biomarkers. Natl Sci. Rev..

[66] Zhang C (2021). Landscape dynamic network biomarker analysis reveals the tipping point of transcriptome reprogramming to prevent skin photodamage. J. Mol. Cell Biol..

[67] Zuo C (2018). Elucidation and analyses of the regulatory networks of upland and lowland ecotypes of switchgrass in response to drought and salt stresses. PLoS One.

[68] Liu, D. et al. Molecular bases of morphologically diffused tumors across multiple cancer types. *Natl Sci. Rev.*10.1093/nsr/nwac177 (2022). 10.1093/nsr/nwac177 PMC9744092 36523564

[69] Yi F, Huang J, Yang L, Xie Y, Xiao G (2017). Automatic extraction of cell nuclei from H&E-stained histopathological images. J. Med. Imaging.

[70] Greenwald NF (2022). Whole-cell segmentation of tissue images with human-level performance using large-scale data annotation and deep learning. Nat. Biotechnol..

[71] Chen, T., Kornblith, S., Norouzi, M. & Hinton, G. A simple framework for contrastive learning of visual representations. In *PMLR* 1597–1607 (2020).

[72] Li, Q., Han, Z. & Wu, X.-M. Deeper insights into graph convolutional networks for semi-supervised learning. In *Thirty-Second AAAI conference on artificial intelligence* (2018).

[73] Veličković, P. et al. Graph attention networks. In *6th International Conference on Learning Representations, ICLR* (2018).

[74] Bahdanau, D., Cho, K. & Bengio, Y. Neural machine translation by jointly learning to align and translate. Preprint at *bioRxiv*10.48550/arXiv.1409.0473 (2014).

[75] Wu SZ (2021). A single-cell and spatially resolved atlas of human breast cancers. Nat. Genet..

[76] Goldman MJ (2020). Visualizing and interpreting cancer genomics data via the Xena platform. Nat. Biotechnol..

[77] Netanely D, Avraham A, Ben-Baruch A, Evron E, Shamir R (2016). Expression and methylation patterns partition luminal-A breast tumors into distinct prognostic subgroups. Breast Cancer Res..

[78] Stuart T (2019). Comprehensive integration of single-cell data. Cell.

[79] Aran D, Sirota M, Butte AJ (2015). Systematic pan-cancer analysis of tumour purity. Nat. Commun..

[80] Russell BC, Torralba A, Murphy KP, Freeman WT (2008). LabelMe: A database and web-based tool for image annotation. Int. J. Comput. Vis..

[81] Bradski, G. & Kaehler, A. *Learning OpenCV: Computer Vision with the OpenCV Library* (O’Reilly Media, 2008).

[82] Handcock, M. S. & Morris, M. *Relative Distribution Methods in the Social Sciences* (Springer Science & Business Media 1999).

[83] Zuo, C. et al. Elucidating tumor heterogeneity from spatially resolved transcriptomics data by multi-view graph collaborative learning. figshare 10.6084/m9.figshare.19880812 (2022). 10.1038/s41467-022-33619-9 PMC9551038 36216831

[84] Zuo, C. et al. Elucidating tumor heterogeneity from spatially resolved transcriptomics data by multi-view graph collaborative learning. *Zenodo*10.5281/zenodo.6052602 (2022). 10.1038/s41467-022-33619-9 PMC9551038 36216831

[85] Guo X (2018). Global characterization of T cells in non-small-cell lung cancer by single-cell sequencing. Nat. Med..

[86] Tang Z, Kang B, Li C, Chen T, Zhang Z (2019). GEPIA2: An enhanced web server for large-scale expression profiling and interactive analysis. Nucleic Acids Res..

