# Supplementary material for: Elucidating tumor heterogeneity from spatially resolved transcriptomics data by multi-view graph collaborative learning
Source: Nat Commun. 2022 Oct 10;13:5962. doi: 10.1038/s41467-022-33619-9 (PMC9551038; doi:10.1038/s41467-022-33619-9)
Supplement: Supplementary file 1 — Supplementary Information [file 41467_2022_33619_MOESM1_ESM.pdf]

Supplementary Materials of

Elucidating tumor heterogeneity from spatially resolved transcriptomics data by multi-view graph collaborative learning

Chunman Zuo[1,2*], Yijian Zhang[3], Chen Cao[4], Jinwang Feng[5], Mingqi Jiao[6], and Luonan Chen[2,6,7,8*]

[1] Institute of Artificial Intelligence, Donghua University, Shanghai 201620, China

[2] Key Laboratory of Systems Biology, Shanghai Institute of Biochemistry and Cell Biology, Center for Excellence in Molecular Cell Science, Chinese Academy of Sciences, Shanghai 200031, China

[3] Department of General Surgery, Xinhua Hospital Affiliated to Shanghai Jiao Tong University School of Medicine, Shanghai 200092, China

[4] School of Biomedical Engineering and Informatics, Nanjing Medical University, Nanjing 211166, China

[5] Key Laboratory of Information Fusion Technology of Ministry of Education, School of Automation, Northwestern Polytechnical University, Xi'an 710072, China

[6] Key Laboratory of Systems Health Science of Zhejiang Province, Hangzhou Institute for Advanced Study, University of Chinese Academy of Sciences, Chinese Academy of Sciences, Hangzhou 310024, China

[7] Guangdong Institute of Intelligence Science and Technology, Hengqin, Zhuhai, Guangdong 519031, China

[8] School of Life Science and Technology, ShanghaiTech University, Shanghai 201210, China

* To whom correspondence should be addressed.

Email: cmzuo@dhu.edu.cn; lnchen@sibs.ac.cn

Supplementary Note 1

Evaluation of proportion of labels for model training

stMVC is robust to the proportion of labels for the training, which was evaluated based on 12 slices of the human DLPFC dataset with annotations from the previous study [1]. Specifically, for each slice, we (i) randomly selected the spots with labels, with the proportion ranging from 0.1 to 0.9 by 0.1, thus generating nine label datasets; (ii) randomly selected 70% of spots as the training set, the labels of which from one of nine datasets were used to supervise the training of stMVC, stMVC-M, semi-AE, and the three SGATE-based single-view models; and (iii) for each model, predicted the cell clusters by the Louvain algorithm, and assessed the influence of the proportion of labels on the model training via clustering accuracy in terms of average silhouette width (ASW) by calculating the closeness of low-dimensional joint-features between spots within each predicted cell cluster (see Evaluation of clustering). Overall, we observed that the clustering accuracy of all models slightly increases with the proportion of labels for the training, and almost all models have a higher accuracy at the training with 70% labels. Hence, we treated 70% as a cutoff to select labels for model training. Additionally, we found that (i) stMVC achieves higher and comparable performance than stMVC-M and SGATE-SLG; (ii) stMVC, stMVC-M, and SGATE-SLG perform better than that by two HSG-based models; (iii) SGATE-HSG performs better than SGATE-HSG-N; and (iv) SGATE-SLG performs better than semi-AE, showing that graph attention mechanism is responsible for capturing data structure (Supplementary Fig.1). Overall, these results indicate the efficiency of stMVC.

Supplementary Methods

Modeling gene expression data by autoencoder-based framework

Regarding gene expression data, we adopting our previous study modeled it as drawn from negative binomial (NB) distribution by an autoencoder-based framework [2]. Specifically, we learned it's d-dimensional features z through an encoder E, and then transformed z into the parameters of NB distribution by corresponding decoder (D_u and D_θ):

$$p(x|z) = \mathrm{NB}(x; u_x, \theta_x) = \mathrm{NB}(x, l_x; D_u(z), D_\theta(z)) \tag{1}$$

$$\mathrm{NB}(x; u_x, \theta_x) = \frac{\Gamma(x + \theta_x)}{\Gamma(\theta_x)\Gamma(\mathrm{x} + 1)} \left(\frac{u_x}{u_x + \theta_x}\right)^x \left(\frac{\theta_x}{\theta_x + u_x}\right)^{\theta_x} \tag{2}$$

where each dimension of u_x and θ_x indicates the mean and dispersion of NB distribution for each gene, and which is simultaneously inferred by D_u by using 'softmax' activation function at the last layer and D_θ, respectively. One-dimensional constant variable l_x calculated by the sum of read counts of all selected genes for each cell, serves as the cell-specific normalized factors.

The training objective of the model is to maximize the marginal likelihood of observed gene expression data, and the loss function is summarized as follows:

$$\log p(x|z) = E_{z \sim p(z|x, E)}(\log p(x|z; D_u, D_\theta)) \tag{3}$$

In this work, each neural network uses batch normalization, 'relu' is regarded as the activation function between two hidden layers, and the Adam optimizer with both a $1e^{-6}$ weight decay and $8e^{-5}$ learning rate is used to minimize the above loss function. In addition, we utilized the autoencoder structure (i.e., [*N*, 1000, 50, 1000, *N*]) to capture the inner structure of gene expression data. Here, for the data from Visium and STARmap, *N* is 2,000 and 1,020, respectively.

Learning representations from RNA-seq data by semi-AE model

To clarify if or not the graph attention mechanism is responsible for capturing the complex data structure, we further extended the usage of AE model described by Modeling gene expression data by autoencoder-based framework to do spot class prediction $Y' = \mathrm{softmax}((W^{(1)}z))$ in a semi-supervised manner from region segmentation, and the loss function of which is summarized as follows:

$$L_{prediction} = \frac{1}{S}\sum_{l=1}^{S}(-\sum_{i=1}^{K} y_i \log(y_i')) \quad (4)$$

where S is the number of labeled spots, K is the number of classes, and y_i and y_i' are the label vector of spot v_i from the region segmentation and the prediction, respectively.

Taken together, the loss function of semi-AE model is summarized as:

$$L_t = \log p(x|z) + \beta L_{pprediction} \quad (5)$$

where β is a parameter used to control the weight of two loss functions, and the default value is 90, at which semi-AE model achieves a better performance in our large-scale experiments.

Statistical model for testing genes enriched in different cell populations

We designed a Fisher's exact test-based measure to check whether or not two genes (or two gene sets) are enriched in different cell populations. Note that the average expression of all genes within a gene set is considered the expression level of the gene set. Specifically, we created the contingency table based on the following two metrics: classification of each cell based on whether or not it expresses gene (set) A or gene (set) B. Fisher's exact test was used to check whether or not cells expressing gene (set) A and cells expressing gene (set) B are correlated. The two genes (or gene sets) are considered from different cell populations if the corresponding $p - value > 0.05$.

Estimation of cell populations for each spot by SpatialDecon

To verify whether or not different cancer cell states (distributed in different spatial locations) are influenced by the infiltrating stromal and immune cells, we adopted a recent deconvolution method SpatialDecon [3] for SRT data to estimate the cell populations of each spot in ovarian and breast cancers. Specifically, for ovarian cancer, we directly utilized the deconvolution result predicted by the developers of SpatialDecon [4], and for each spot where the proportion of tumor cells was less than 95%, treated the predicted two cell types with the highest proportions as its infiltrating stromal and immune cells, otherwise, considered it as pure tumor cells. In addition, we followed the tutorial for processing breast cancer to estimate the cell populations of different stromal and immune cells in breast cancer [5], and for each spot, considered the two cell types with the highest proportions as its infiltrating stromal and immune cells.

Supplementary Figures

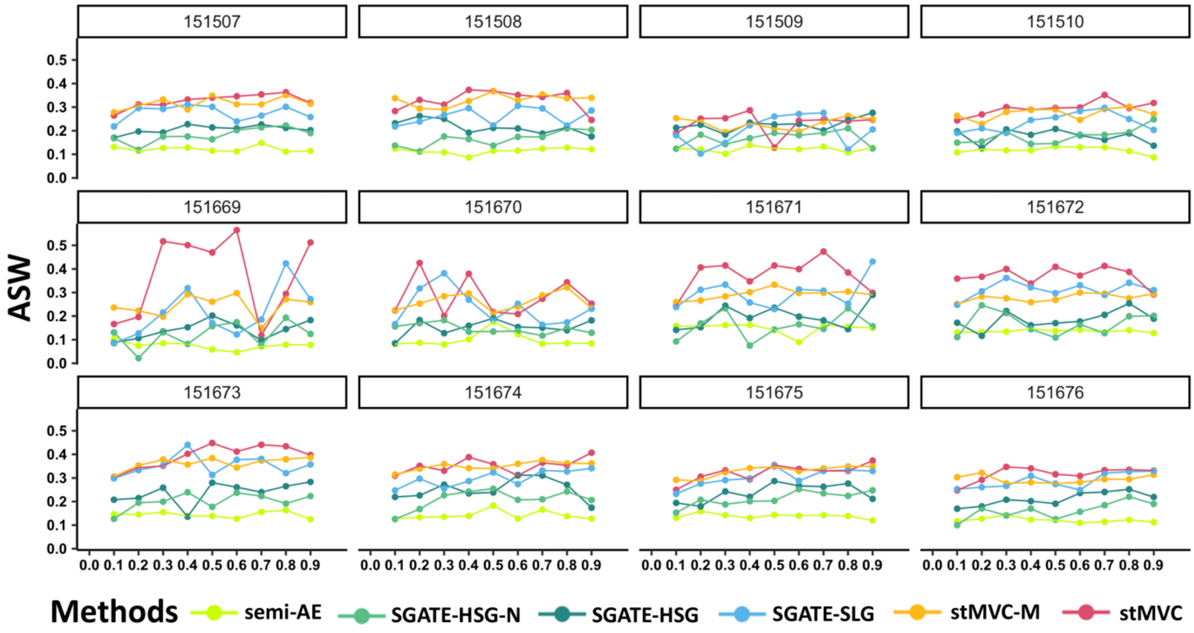

Supplementary Figure 1. Evaluation of the proportion of labels used to train the stMVC, stMVC-M, semi-AE, and the three SGATE-based single-view models by clustering accuracy in terms of ASW on the 12 slices of human DLPFC dataset. Each color indicates one method. The X-axis indicates the proportion of labels for the training of the model. Source data are provided as a Source Data file.

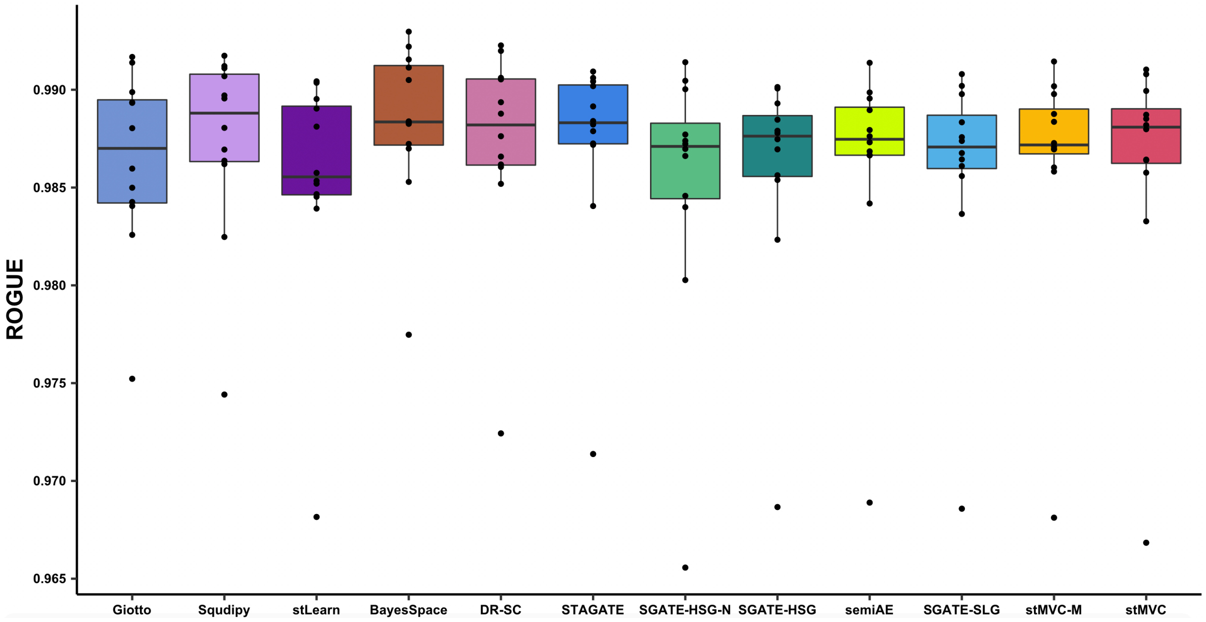

Supplementary Figure 2. Boxplot of ROGUE to assess the transcriptome similarity between spots within each predicted cluster for $n = 12$ slices of the DLPFC dataset. For each slice, the mean of ROGUE values of all predicted clusters indicates its ROGUE value. For each boxplot, the center line, box limits and whiskers separately indicate the median, upper and lower quartiles and $1.5 \times$ interquartile range. Source data are provided as a Source Data file.

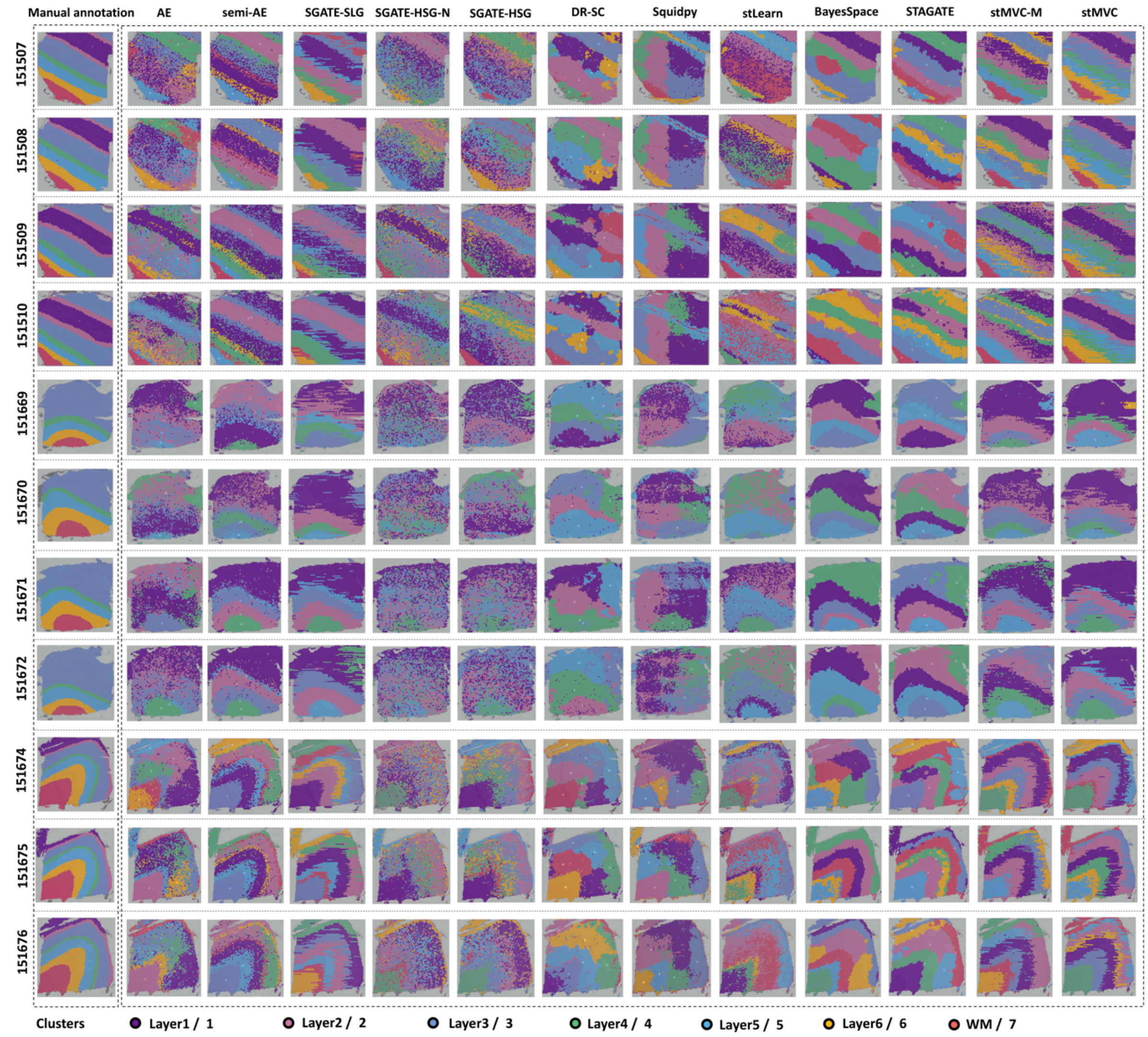

Supplementary Figure 3. Spatial domains were detected by AE, semi-AE, the three SGATE-based single-view models, DR-SC, Squidpy, stLearn, BayesSpace, STAGATE, stMVC-M, and stMVC, where we also provide manual annotation as a comparison, on 11 slices of the DLPFC dataset. Source data are provided as a Source Data file.

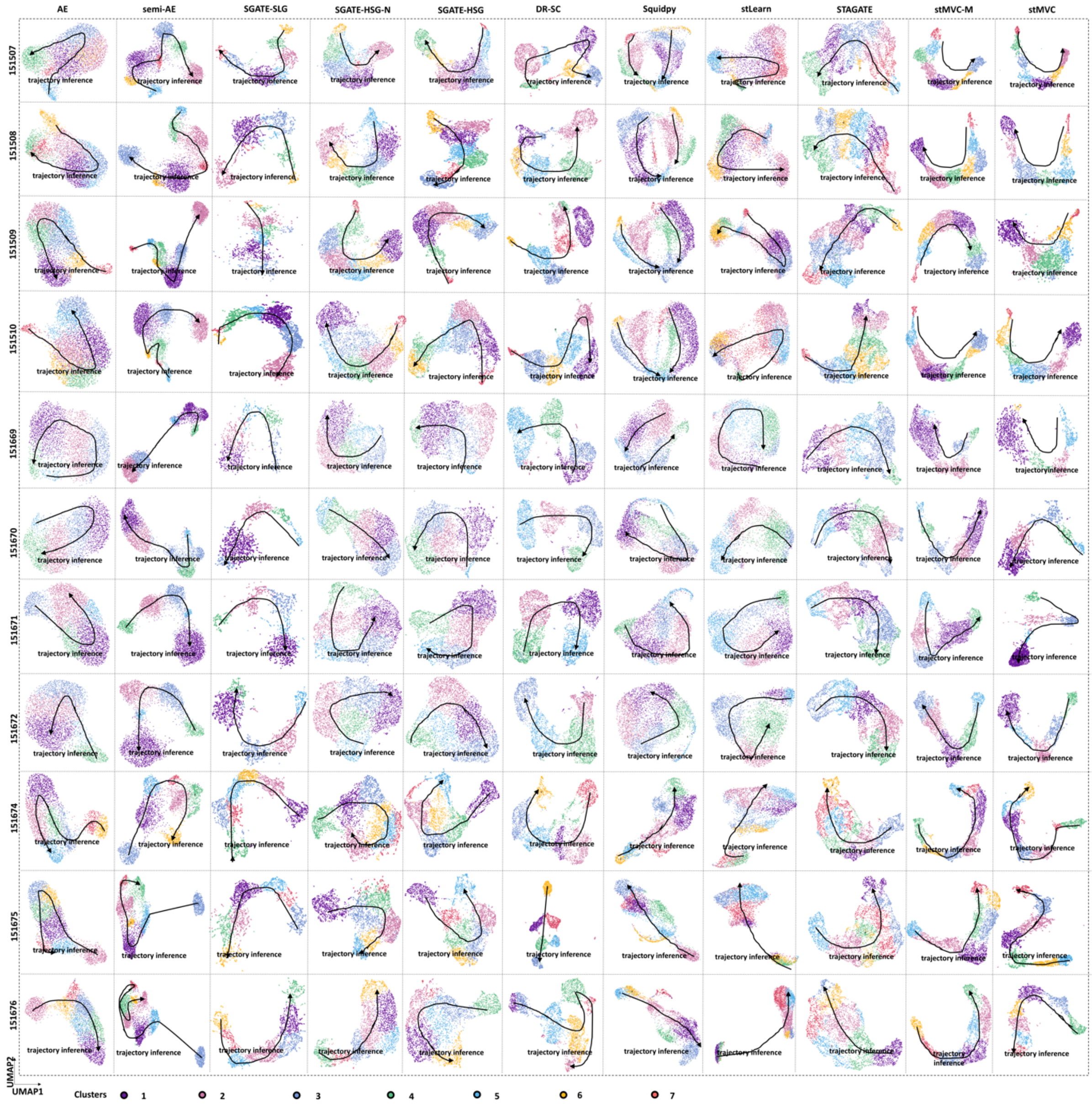

Supplementary Figure 4. Scatter plot of the two-dimensional UMAP extracted from the latent features by AE, semi-AE, the three SGATE-based single-view models, DR-SC, Squidpy, stLearn, STAGATE, stMVC-M, and stMVC, on 11 slices of the human DLPFC dataset. For each method on each slice, the predicted clusters and their colors are the same as Supplementary Fig.3. The inferred trajectory between different clusters is consistent with Fig.2d. Source data are provided as a Source Data file.

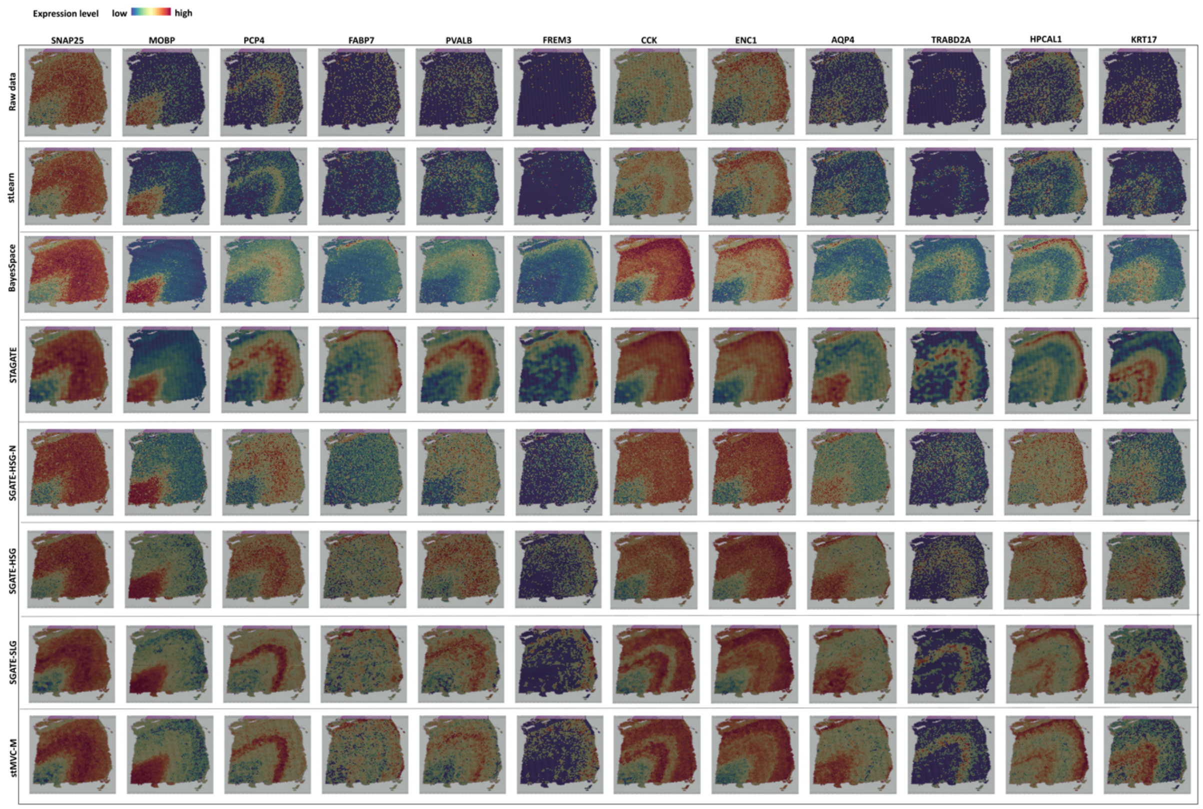

Supplementary Figure 5. Spatial expression of layer-specific genes [1]: *SNAP25*, *MOBP*, *PCP4*, *FABP7*, *PVALB*, *FREM3*, *CCK*, *ENC1*, *AQP4*, *TRABD2A*, *HPCA1*, and *KRT17* for slice 151673 data denoised by stLearn, BayesSpace, STAGATE, the three SGATE-based single-view models, and stMVC-M, respectively, where we also provide raw data as a comparison. Source data are provided as a Source Data file.

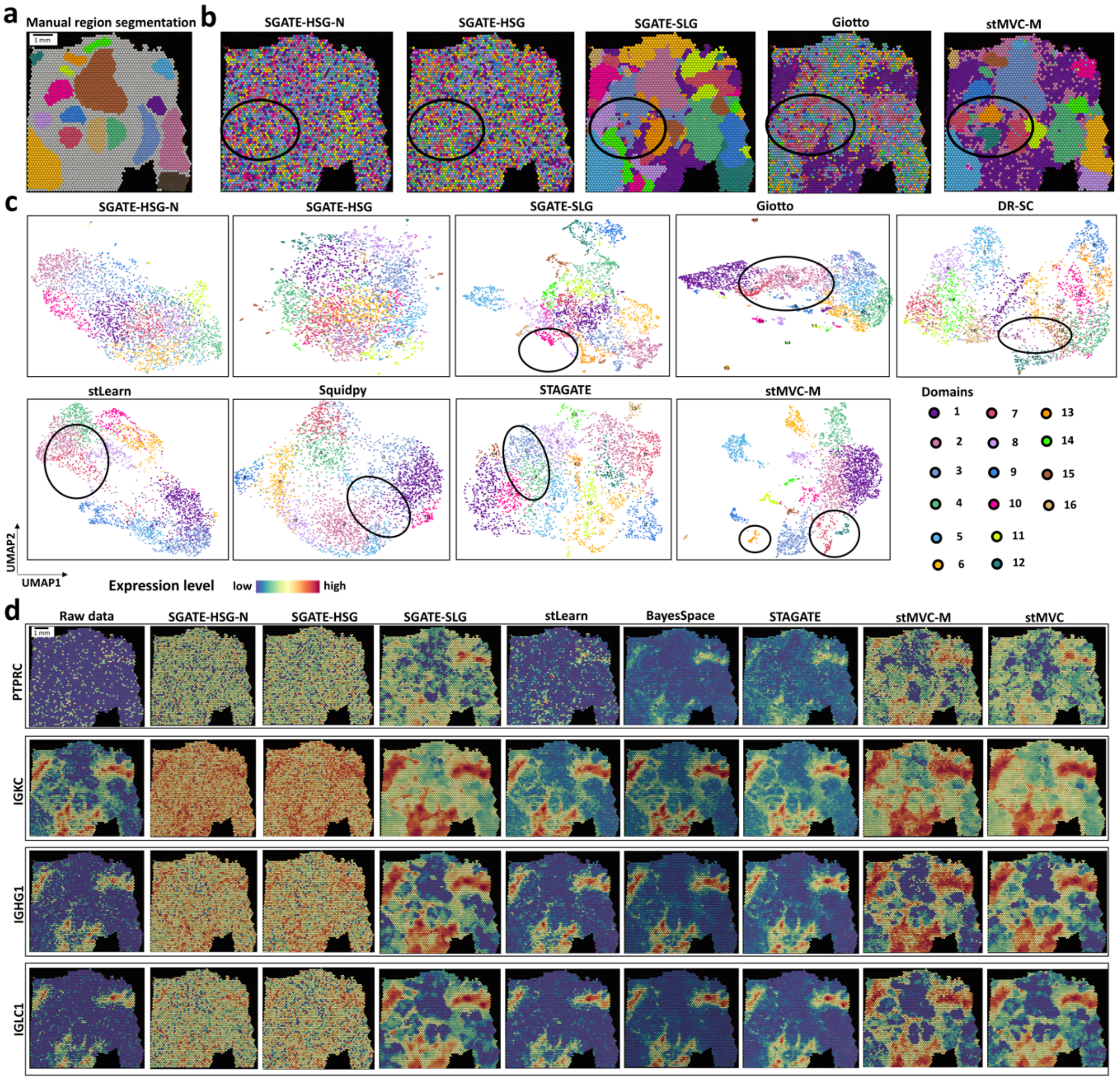

Supplementary Figure 6. Method comparisons on the ovarian cancer sample. **a** Manual segmentation with 18 distinct regions. Each region is indicated by one color. **b** Spatial clustering by the three SGATE-based single-view models, Giotto, and stMVC-M, respectively. Each domain is indicated by one color. **c** UMAP visualization of the latent features by the three SGATE-based single-view models, Giotto, DR-SC, stLearn, Squidpy, STAGATE, and stMVC-M, respectively. For each method, the predicted clusters and their colors are the same as **b** and Fig.3b. **d** Spatial expression of genes for immune-related markers: *PTPRC*, *IGKC*, *IGHG1*, *IGLC1* for the data denoised by the three SGATE-based single-view models, BayesSpace, stLearn, STAGATE, stMVC-M, and stMVC, respectively, where we also provide raw data as a comparison. Source data are provided as a Source Data file.

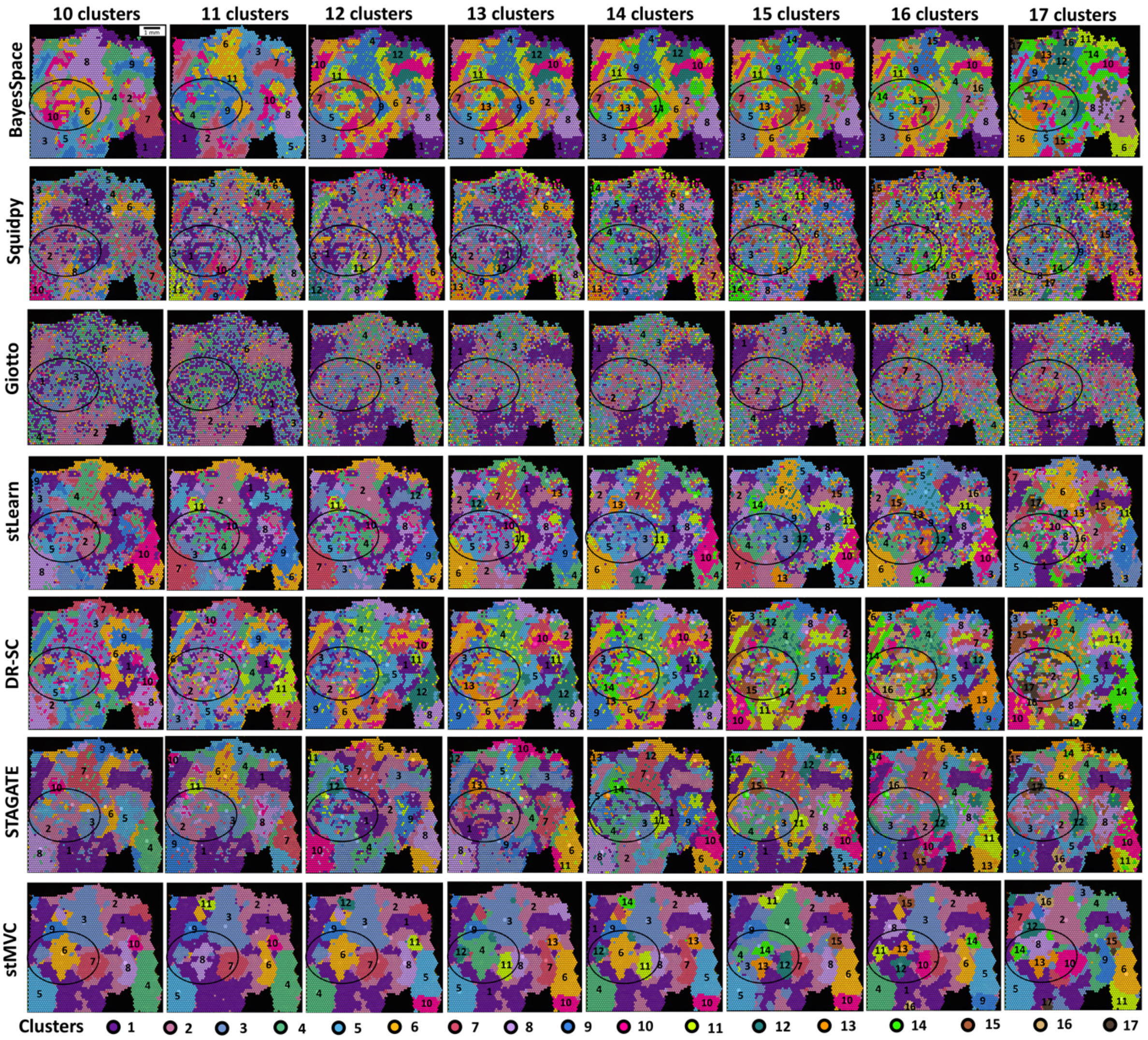

Supplementary Figure 7. Comparison of spatial clustering of human ovarian cancer sample by BayesSpace, Squidpy, Giotto, stLearn, DR-SC, STAGATE, and stMVC, where the number of clusters ranges from 10 to 17. Source data are provided as a Source Data file.

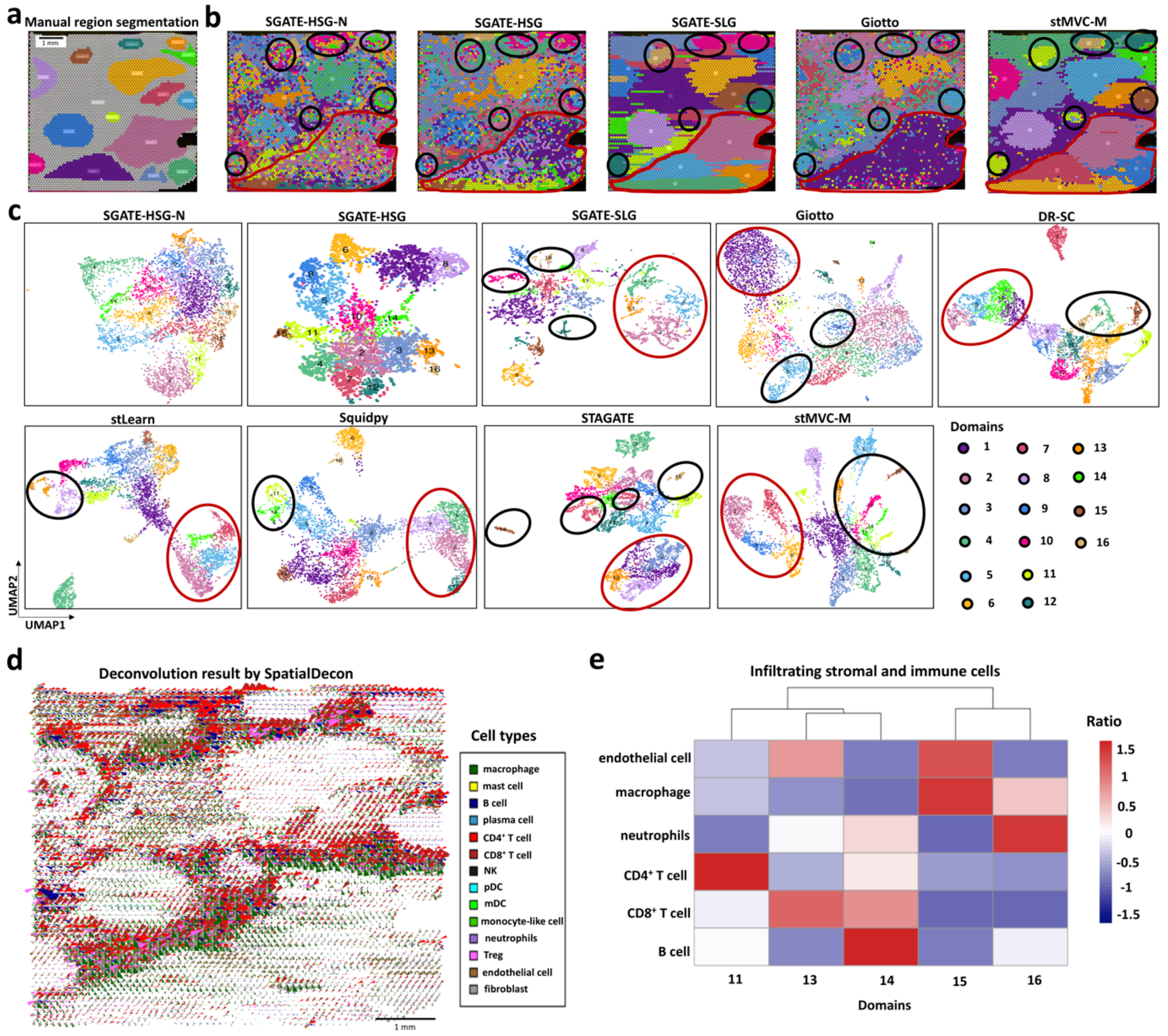

Supplementary Figure 8. Method comparisons on the breast cancer sample. **a** Manual segmentation with 16 distinct regions. Each color indicates one region. **b** Spatial clustering by the three SGATE-based single-view models, Giotto, and stMVC-M, respectively. Each domain is indicated by one color. **c** UMAP visualization of the latent features by the three SGATE-based single-view models, Giotto, DR-SC, stLearn, Squidpy, STAGATE, and stMVC-M, respectively. For each method, the predicted clusters and their colors are the same as **b** and Fig.3i. **d** Abundance estimate of 14 cell types in the microenvironment segments of the breast cancer by SpatialDecon. Wedge size is proportional to estimated cell counts. NK: natural killer cell. pDC: plasmacytoid dendritic cell. mDC: myeloid dendritic cell. **e** The enrichment of six distinct cell types in each domain compared to the total distribution of six cell types in five domains. The ratio is calculated by the chi-square test, which is the same with Fig.3f. The larger the ratio, the more cells are enriched in the domain. The proportion patterns of infiltrating stromal and immune cells between domains 13 and 14, and between domains 15 and 16 are more similar than those with other domains. Source data are provided as a Source Data file.

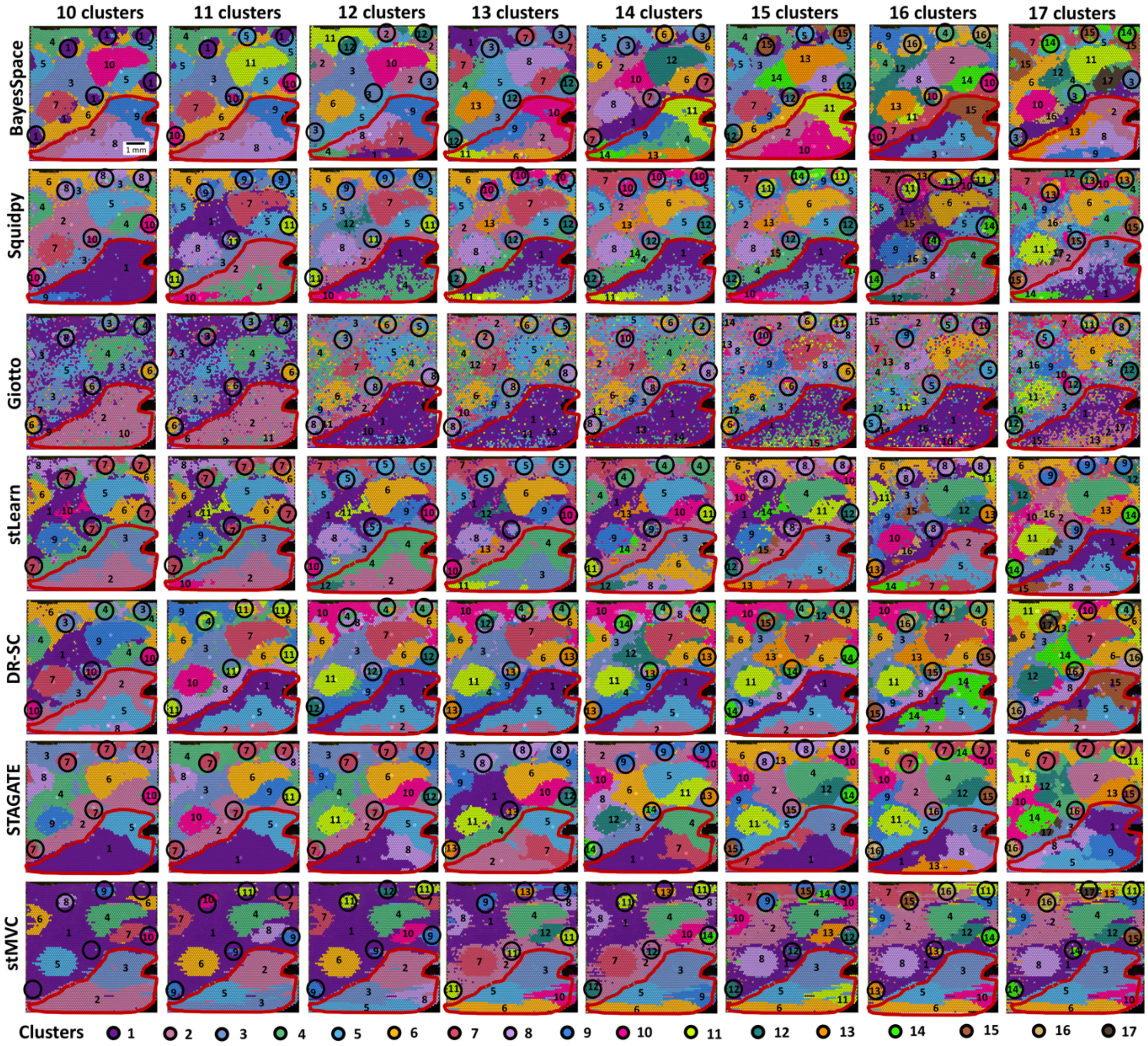

Supplementary Figure 9. Comparison of spatial clustering of human breast cancer sample by BayesSpace, Squidpy, Giotto, stLearn, DR-SC, STAGATE, and stMVC, where the number of clusters ranges from 10 to 17. Source data are provided as a Source Data file.

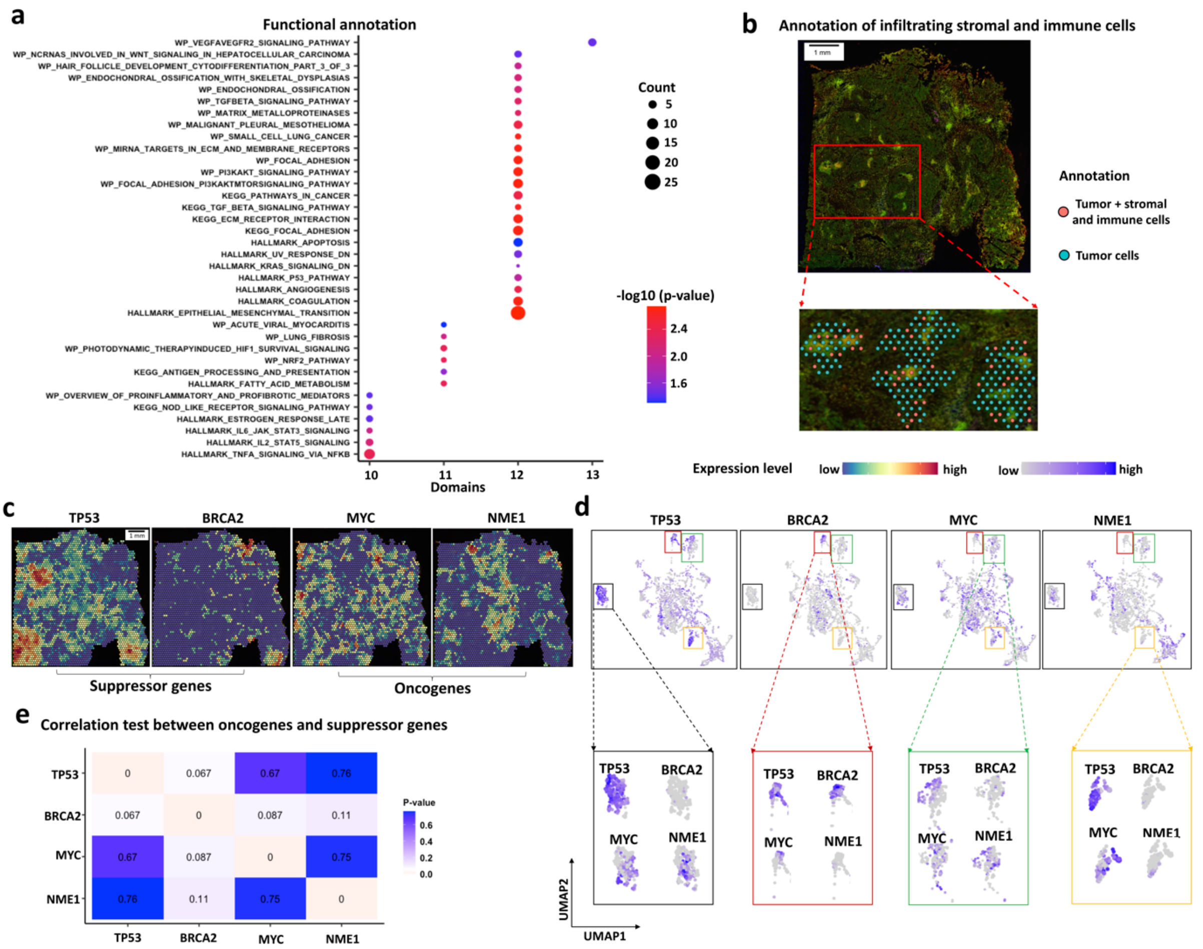

Supplementary Figure 10. Annotation of cancer regions in the ovarian cancer sample by stMVC. **a** Gene function enrichment analysis of SVGs in each of four domains by R package clusterProfiler [6]. The functional gene sets were downloaded from MSigDB database (https://www.gsea-msigdb.org/gsea/msigdb/) [7]. The enrichment test was conducted by GSEA with Kolmogorov-Smirnov statistics. The $p - value$ is estimated by one-sided tests without adjustment for multiple comparisons. The dot size and color indicate the count and $p - value$ of each function in each domain, respectively. **b** Annotation of four interested cancer regions by infiltrating stromal and immune cells. The cell populations for each spot were estimated by SpatialDecon (see **Estimation of cell populations for each spot by SpatialDecon**). Note that pure tumor cells and the tumor cells with infiltrating stromal and immune cells are indicated by different colors. **c** Spatial expression of tumor suppressor genes (i.e., *TP53* and *BRCA2*) and oncogenes (i.e., *MYC* and *NME1*) in ovarian cancer sample. **d** The UMAP plot of the expression levels of *TP53*, *BRCA2*, *MYC*, and *NME1* in ovarian cancer sample. **e** Correlation test for the expression levels of tumor suppressor genes (*TP53* and *BRCA2*) and oncogenes (*MYC* and *NME1*) by Fisher's exact test with the two-sided. Here, the value and color indicate $p - value$. Source data are provided as a Source Data file.

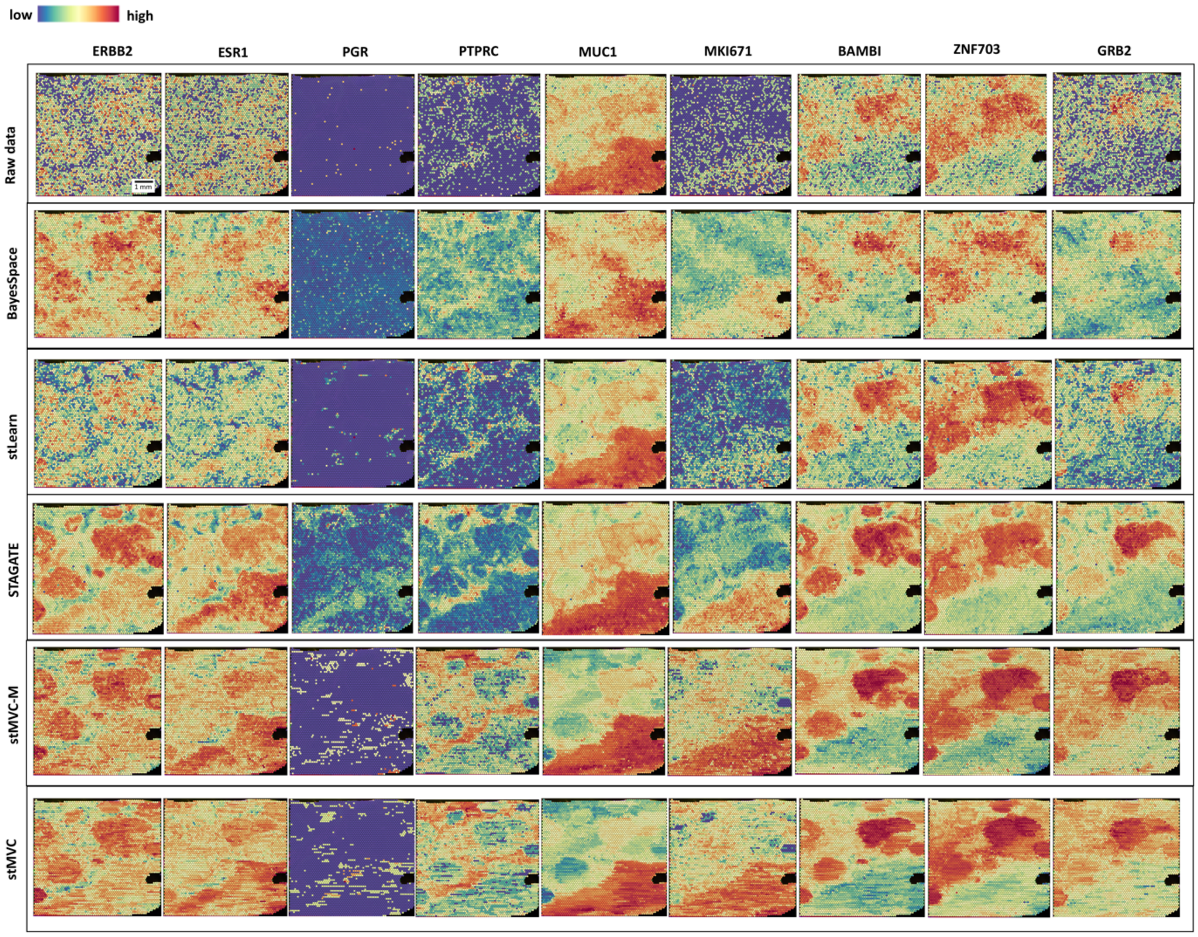

Supplementary Figure 11. Spatial expression of genes for indicative markers: *ERBB2*, *ESR1*, *PGR*, immune genes: *PTPRC*, and tumor progression genes: *MUC1*, *MKI67*, *BAMBI*, *ZNF703*, and *GRB2* for the data denoised by BayesSpace, stLearn, STAGATE, stMVC-M, and stMVC, respectively, where we also provide raw data as a comparison. Source data are provided as a Source Data file.

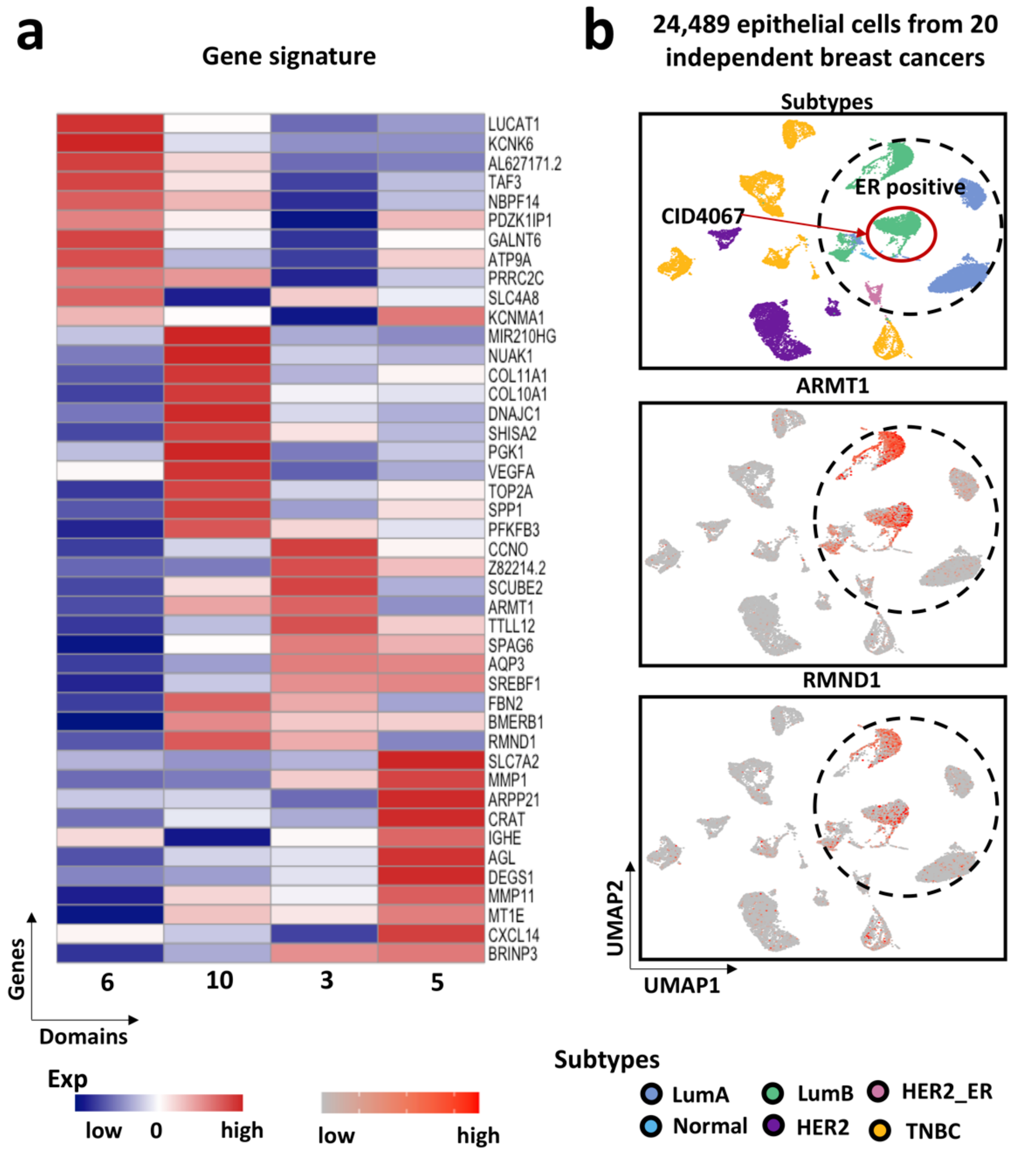

Supplementary Figure 12. Data analysis in breast cancer sample. **a** Heatmap of the gene expression of signature genes for four domains enriched in the ER^+ invasive carcinoma region by stMVC. Rows and columns indicate signature genes and different domains, respectively. **b** UMAP visualization of independent scRNA-seq data of 24,489 epithelial cells from 20 breast cancer patients, as well as the expression levels of *ARMT1* and *RMND1*. Each color indicates one subtype of breast cancer determined by the status of ER, PR, and HER2 (up panel). Source data are provided as a Source Data file.

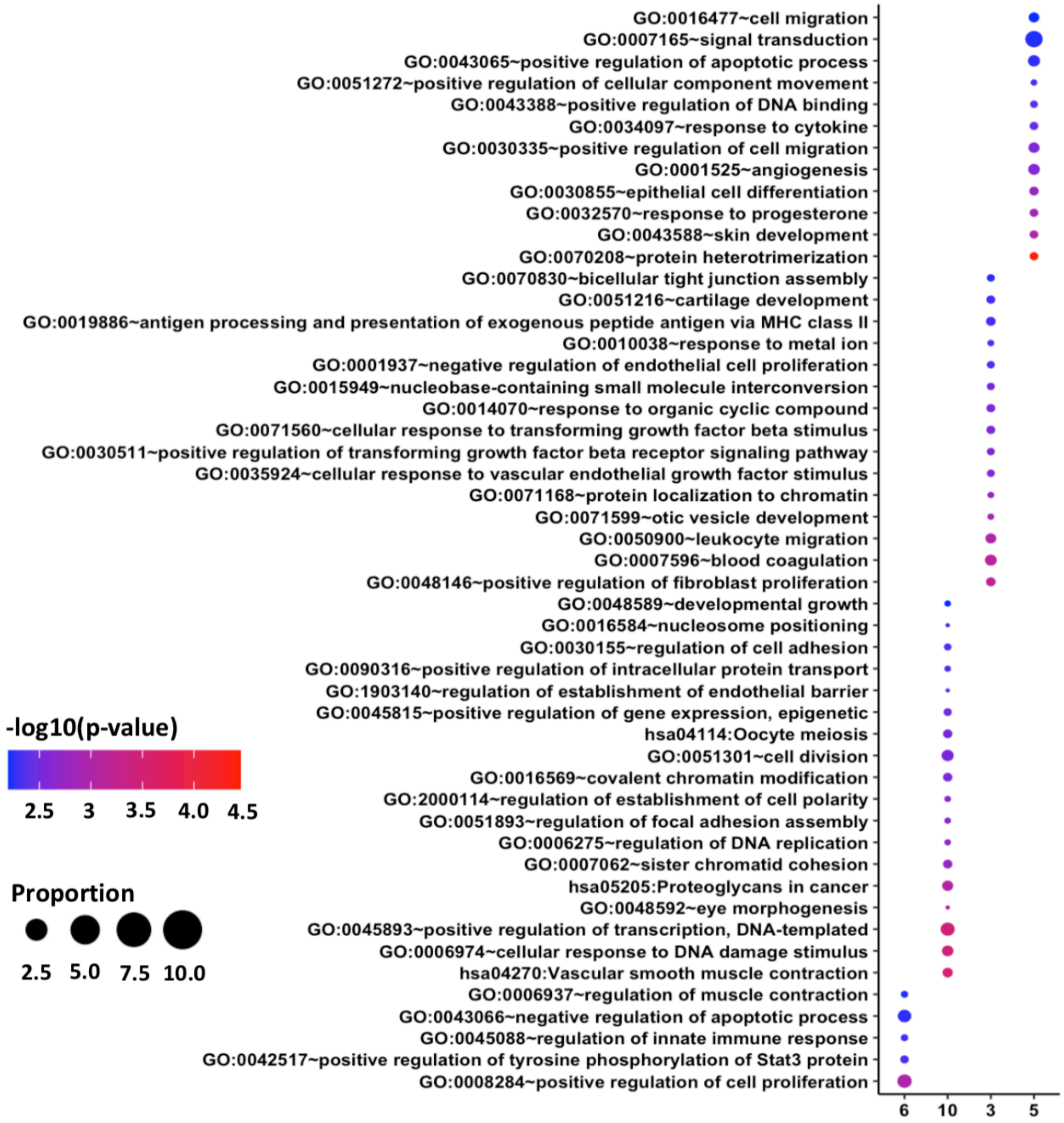

Supplementary Figure 13. Gene function enrichment analysis of SVGs in four domains enriched in the ER^+ invasive carcinoma region from stMVC by DAVID with the hypergeometric test (one-sided) [8]. No adjustment for multiple comparisons was made. Dot size and color indicate the percentage and $p-value$ of each function in each domain, respectively. Source data are provided as a Source Data file.

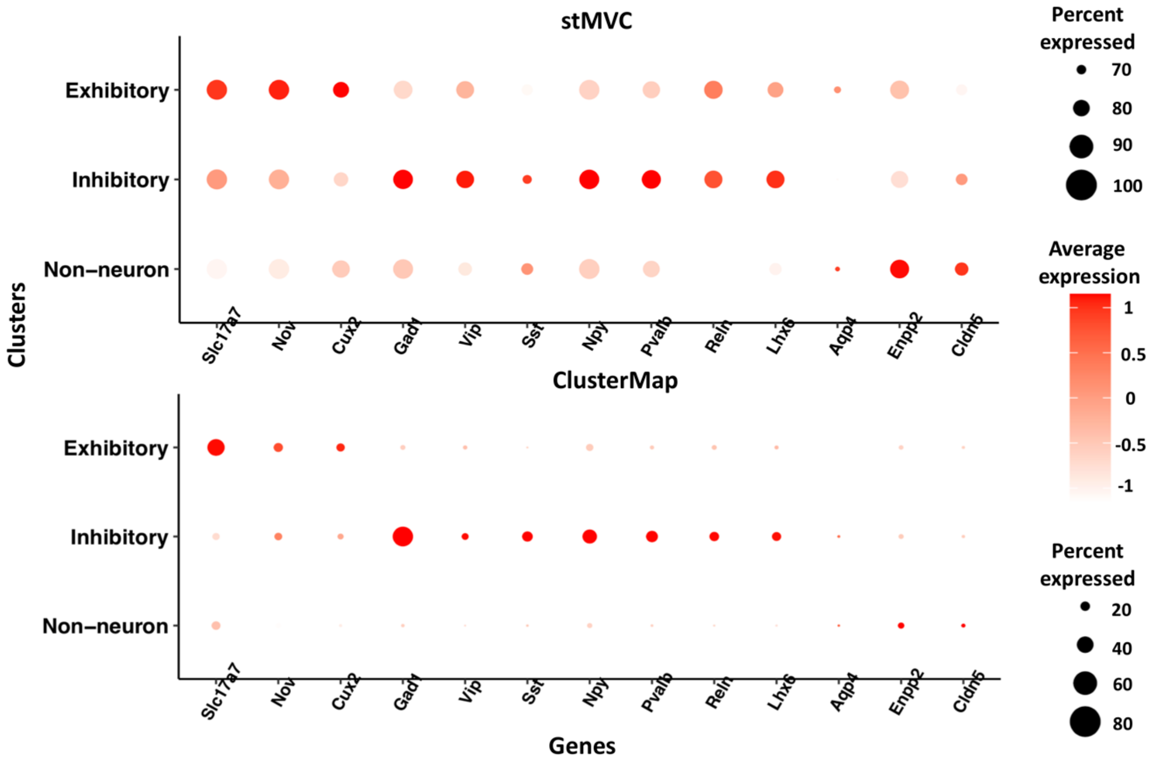

Supplementary Figure 14. Dot plot showing the expression levels of marker genes for different cell clusters predicted by stMVC and ClusterMap, respectively. Note that *Slc17a7*, *Nov*, and *Cux2* for excitatory neuron, *Gad1*, *Vip*, *Sst*, *Npy*, *Pvalb*, *Reln*, and *Lhx6* for inhibitory neuron, and *Aqp4*, *Enpp2*, and *Cldn5* for non-neuronal cell from the previous study [9]. Source data are provided as a Source Data file.

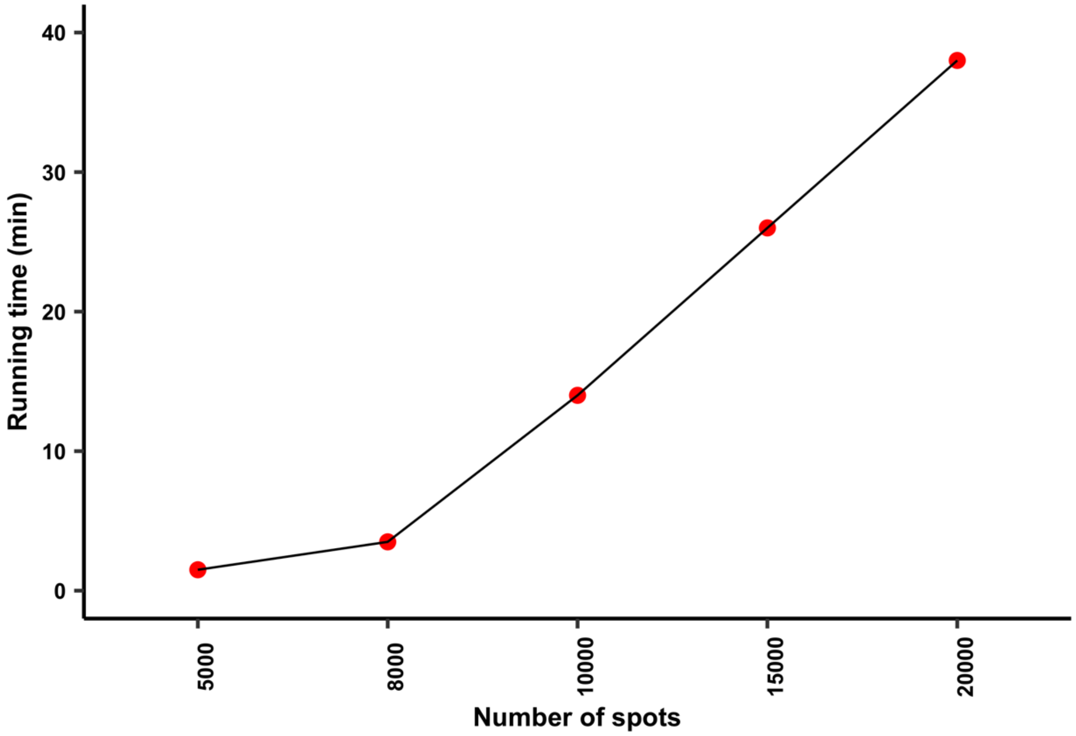

Supplementary Figure 15. Comparison of running time for the training of stMVC model on the different number of spots by subsampling from the human DLPFC datasets. The experiments were tested on a GPU server with two NVIDIA Tesla V100 GPU addressing 64GB. Source data are provided as a Source Data file.

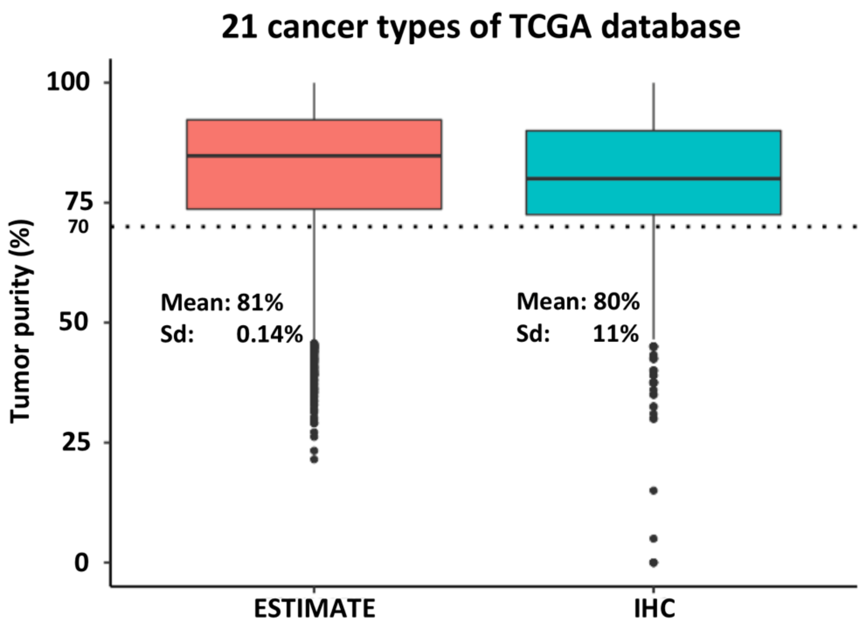

Supplementary Figure 16. Boxplot of the tumor purity of 21 cancer types from TCGA database. The tumor purity predicted by the computational methods (i.e., ESTIMATE and IHC) was downloaded from the previous research [10]. The average tumor purities estimated by ESTIMATE (across 7,737 samples) and IHC (across 9,327 samples) are $81 \pm 14\%$ and $80 \pm 11\%$, respectively. The average tumor purity is larger than 80%, and the tumor purity of more than 80% of samples is greater than 70%. For each boxplot, the center line, box limits and whiskers separately indicate the median, upper and lower quartiles and $1.5 \times$ interquartile range. Source data are provided as a Source Data file.

Supplementary Tables

Supplementary Table 1. The signature genes used for defining four different domains in ovarian cancer sample.

Domains	Signature genes
Domain 10	*S100A9*, *SERPINA3*, *PDZK1IP1*, *PNOC*, *PTX3*, *CXCL8*, *DEFB1*, *DDIT4*, *CCL20*, *PI3*, *LAMA3*
Domain 11	*SPP1*, *VEGFA*, *SLC2A1*, *DDIT4*, *PGK1*, *ENO2*, *PI3*, *SLC2A3*, *MT2A*, *ADM*, *HSPA6*
Domain 12	*IGFBP5*, *MMP10*, *GPRC5A*, *GJB2*, *IGFL2*, *OAS2*, *LAMA3*, *COL10A1*, *TIMP3*, *COMP*, *LAMC2*
Domain 13	*MMP10*, *NEDD9*, *EMP1*, *S100A4*, *BCAT1*, *TRIB2*, *PTX3*, *LRIG1*, *DPYSL2*, *SLC27A6*, *BMP8A*

Supplementary Table 2. The layer-specific genes for human DLPFC dataset.

Genes	*SNAP25*, *MOBP*, *PCP4*, *FABP7*, *PVALB*, *CCK*, *ENC1*, *AQP4*, *TRABD2A*, *HPCAL1*, *FREM3*, *KRT17*

References

1 Maynard, K. R. *et al.* Transcriptome-scale spatial gene expression in the human dorsolateral prefrontal cortex. *Nature neuroscience* **24**, 425-436 (2021).

2 Zuo, C., Dai, H. & Chen, L. Deep cross-omics cycle attention model for joint analysis of single-cell multi-omics data. *Bioinformatics* **37**, 4091-4099 (2021).

3 Danaher, P. *et al.* Advances in mixed cell deconvolution enable quantification of cell types in spatial transcriptomic data. *Nat Commun* **13**, 1-13 (2022).

4 Nanostring-Biostats. Analysis of example Visium data. *GitHub* https://github.com/Nanostring-Biostats/SpatialDecon-manuscript-analyses (2022).

5 Griswold, M. Use of SpatialDecon in a Spatial Transcriptomics dataset. *bioconductor* https://bioconductor.org/packages/release/bioc/vignettes/SpatialDecon/inst/doc/SpatialDecon_vignette_ST.html (2022).

6 Yu, G., Wang, L.-G., Han, Y. & He, Q.-Y. clusterProfiler: an R package for comparing biological themes among gene clusters. *Omics: a journal of integrative biology* **16**, 284-287 (2012).

7 Subramanian, A. *et al.* Gene set enrichment analysis: a knowledge-based approach for interpreting genome-wide expression profiles. *Proceedings of the National Academy of Sciences* **102**, 15545-15550 (2005).

8 Dennis, G. *et al.* DAVID: database for annotation, visualization, and integrated discovery. *Genome Biol* **4**, 1-11 (2003).

9 Tasic, B. *et al.* Adult mouse cortical cell taxonomy revealed by single cell transcriptomics. *Nature neuroscience* **19**, 335-346 (2016).

10 Aran, D., Sirota, M. & Butte, A. J. Systematic pan-cancer analysis of tumour purity. *Nat Commun* **6**, 1-12 (2015).
