# Supplementary material for: Elucidating tumor heterogeneity from spatially resolved transcriptomics data by multi-view graph collaborative learning
Source: Nat Commun. 2022 Oct 10;13:5962. doi: 10.1038/s41467-022-33619-9 (PMC9551038; doi:10.1038/s41467-022-33619-9)
Supplement: Supplementary file 2 — Reporting Summary [file 41467_2022_33619_MOESM2_ESM.pdf]

nature portfolio

Corresponding author(s): Luonan Chen and Chunman Zuo

Last updated by author(s): Sep 4, 2022

Reporting Summary

Nature Portfolio wishes to improve the reproducibility of the work that we publish. This form provides structure for consistency and transparency in reporting. For further information on Nature Portfolio policies, see our Editorial Policies and the Editorial Policy Checklist.

Statistics

For all statistical analyses, confirm that the following items are present in the figure legend, table legend, main text, or Methods section.

n/a	Confirmed	
☐	☒	The exact sample size (*n*) for each experimental group/condition, given as a discrete number and unit of measurement
☐	☒	A statement on whether measurements were taken from distinct samples or whether the same sample was measured repeatedly
☐	☒	The statistical test(s) used AND whether they are one- or two-sided<br>*Only common tests should be described solely by name; describe more complex techniques in the Methods section.*
☒	☐	A description of all covariates tested
☐	☒	A description of any assumptions or corrections, such as tests of normality and adjustment for multiple comparisons
☐	☒	A full description of the statistical parameters including central tendency (e.g. means) or other basic estimates (e.g. regression coefficient) AND variation (e.g. standard deviation) or associated estimates of uncertainty (e.g. confidence intervals)
☐	☒	For null hypothesis testing, the test statistic (e.g. *F*, *t*, *r*) with confidence intervals, effect sizes, degrees of freedom and *P* value noted<br>*Give P values as exact values whenever suitable.*
☒	☐	For Bayesian analysis, information on the choice of priors and Markov chain Monte Carlo settings
☒	☐	For hierarchical and complex designs, identification of the appropriate level for tests and full reporting of outcomes
☒	☐	Estimates of effect sizes (e.g. Cohen's *d*, Pearson's *r*), indicating how they were calculated

Our web collection on statistics for biologists contains articles on many of the points above.

Software and code

Policy information about availability of computer code

Data collection	No software was used.
Data analysis	stMVC is implemented based on python 3.6.12 and R 4.0.0. Other tools and packages used in the data analysis include: numpy 1.19.2, pandas 1.1.5, scipy 1.5.2, scikit-learn 0.23.3, torch 1.6.0, tqdm 4.55.0, scanpy 1.6.0, PIL 9.1.0, seaborn 0.11.2, sklearn 1.0.2, matplotlib 3.5.2, glob2, anndata 0.8.0, argparse 1.1, json 2.0.9, R 4.0.0, Seurat v4, ggplot2 3.3.5, monocle 2.10.1, SpatialDecon 1.6.0, DR-SC 2.9, STAGATE 1.0.1, cv2 (OpenCV) 4.5.5, labelme 5.0.1, ClusterMap 0.0.1, Squidpy 1.2.2, stLearn 0.3.1, BayesSpace 1.1.4, and Giotto 1.0.4. The codes are available at Zenodo https://zenodo.org/record/6052602 [Ref. 84]. The stMVC tool will be maintained and updated at https://github.com/cmzuo11/stMVC.

For manuscripts utilizing custom algorithms or software that are central to the research but not yet described in published literature, software must be made available to editors and reviewers. We strongly encourage code deposition in a community repository (e.g. GitHub). See the Nature Portfolio guidelines for submitting code & software for further information.

Data

Policy information about availability of data

All manuscripts must include a data availability statement. This statement should provide the following information, where applicable:

- Accession codes, unique identifiers, or web links for publicly available datasets
- A description of any restrictions on data availability
- For clinical datasets or third party data, please ensure that the statement adheres to our policy

The raw count matrix, histological image, and spatial location data for both human ovarian and breast cancer samples are publicly available at the 10X Genomics Website (https://support.10xgenomics.com/spatial-gene-expression/datasets). The raw count matrix, image, and spatial location data for 12 slices of human DLPFC dataset are available from the package spatialLIBD (http://spatial.libd.org/spatialLIBD/) 26. The DAPI image and RNA clusters per cell for mouse primary visual cortex 1020-gene sample are publicly available from GitHub link of ClusterMap (https://github.com/wanglab-broad/ClusterMap). The additional scRNA-seq data of 20 human breast cancers and one human ovarian cancer are publicly available from Gene Expression Omnibus database under accession code GSE176078 (https://www.ncbi.nlm.nih.gov/geo/query/acc.cgi?acc=GSE176078) and EMBL-EBI database under accession code E-MTAB-8859 (https://www.ebi.ac.uk/arrayexpress/experiments/E-MTAB-8859/) respectively. The bulk RNA-seq and clinical data from the TCGA database are at the Xena platform (https://xenabrowser.net/datapages/). The functional gene sets are at MSigDB database (https://www.gsea-msigdb.org/gsea/msigdb/). Source data are available at figshare [Ref. 83].

Human research participants

Policy information about studies involving human research participants and Sex and Gender in Research.

Reporting on sex and gender	Not applicable.
Population characteristics	Not applicable.
Recruitment	Not applicable.
Ethics oversight	Not applicable.

Note that full information on the approval of the study protocol must also be provided in the manuscript.

Field-specific reporting

Please select the one below that is the best fit for your research. If you are not sure, read the appropriate sections before making your selection.

☒ Life sciences ☐ Behavioural & social sciences ☐ Ecological, evolutionary & environmental sciences

For a reference copy of the document with all sections, see nature.com/documents/nr-reporting-summary-flat.pdf

Life sciences study design

All studies must disclose on these points even when the disclosure is negative.

Sample size	No sample size was calculated. All the data used here were downloaded from the public databases, and used to check the ability of stMVC. Here, we utilized (i) the human DLPFC dataset (with substantial known information such as cell annotation, layer-specific marker genes, and so on) from the Visium platform (a sequencing-based technology) to validate the functionality of stMVC in detecting tissue structure, inferring trajectory relationships, and data denoising; (ii) used the human ovarian and breast cancers Visium platform to validate that stMVC can identify more different cell-states (missed by other computational methods) that are distributed from different positions in the tumor; and (iii) used the mouse primary visual cortex (V1) 1020-gene dataset from the STARmap platform to support that stMVC can be applied to gain novel insights from imaging-based spatial transcriptomics technology. Hence, it is sufficient to demonstrate the ability and functionality of stMVC.
Data exclusions	We performed quality control and gene selection for spatial transcriptomics data based on the established standards in the field.
Replication	All temps on the replication were successful and can be independently performed.
Randomization	The allocation was random.
Blinding	All data used here are available from previous research or public database, hence, it is impossible for blinding the investigations in analyzing data by our designed computational algorithms.

Reporting for specific materials, systems and methods

We require information from authors about some types of materials, experimental systems and methods used in many studies. Here, indicate whether each material, system or method listed is relevant to your study. If you are not sure if a list item applies to your research, read the appropriate section before selecting a response.

Materials & experimental systems

n/a	Involved in the study
☒	☐ Antibodies
☒	☐ Eukaryotic cell lines
☒	☐ Palaeontology and archaeology
☒	☐ Animals and other organisms
☒	☐ Clinical data
☒	☐ Dual use research of concern

Methods

n/a	Involved in the study
☒	☐ ChIP-seq
☒	☐ Flow cytometry
☒	☐ MRI-based neuroimaging
